# Towards Routine Condensed Phase Simulations with Delta-Learned Coupled Cluster Accuracy: Application to Liquid Water

**DOI:** 10.1021/acs.jctc.5c01377

**Published:** 2025-11-07

**Authors:** Niamh O’Neill, Benjamin X. Shi, William J. Baldwin, William C. Witt, Gábor Csányi, Julian D. Gale, Angelos Michaelides, Christoph Schran

**Affiliations:** † Yusuf Hamied Department of Chemistry, 2152 University of Cambridge, Lensfield Road, Cambridge CB2 1EW, U.K.; ‡ Cavendish Laboratory, Department of Physics, University of Cambridge, Cambridge CB3 0HE, U.K.; § Lennard-Jones Centre, University of Cambridge, Trinity Ln, Cambridge CB2 1TN, U.K.; ∥ Initiative for Computational Catalysis, Flatiron Institute, 160 fifth Avenue, New York, New York 10010, United States; ⊥ Department of Engineering, University of Cambridge, Cambridge CB3 0HE, U.K.; # Harvard John A. Paulson School of Engineering and Applied Sciences, 1649 Harvard University, Cambridge, Massachusetts 02138, United States; ∇ School of Molecular and Life Sciences, Curtin University, PO Box U1987, Perth, Western Australia 6845, Australia

## Abstract

Simulating liquid water to an accuracy that matches its wealth of available experimental data requires both precise electronic structure methods and reliable sampling of nuclear (quantum) motion. This is challenging because applying the electronic structure method of choice, coupled cluster theory with single, double, and perturbative triple excitations [CCSD­(T)] to condensed phase systems, is currently limited by its computational cost and complexity. Recent *tour de force* efforts have demonstrated that this accuracy can indeed bring simulated liquid water into close agreement with experiment using machine learning potentials (MLPs). However, achieving this remains far from routine, requiring large datasets and significant computational cost. In this work, we introduce a practical approach that combines developments in MLPs with local correlation approximations to enable routine CCSD­(T)-level simulations of liquid water. When combined with nuclear quantum effects, we achieve agreement with experiments for structural and transport properties. Importantly, the approach also handles constant-pressure simulations, enabling MLP-based CCSD­(T) models to predict isothermal–isobaric bulk properties, such as water’s density maximum, in close agreement with experiment. Encompassing tests across electronic structure, datasets, and MLP architecture, this work provides a practical blueprint towards routinely developing CCSD­(T)-based MLPs for the condensed phase.

## Graphical abstract

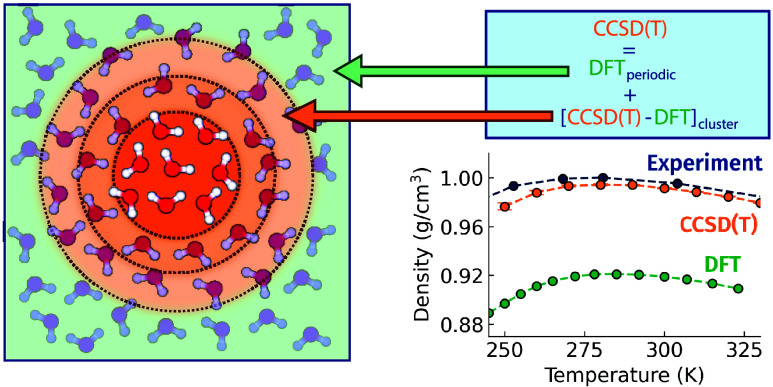

## 1 Introduction

Water is a widely studied system of fundamental scientific and technological importance. Its extensive body of experimental data makes it a fertile testing ground for evaluating new developments in atomistic simulation methods. More broadly, it is the prototypical “condensed phase” system, serving as a bridge between gas phase molecules and solid-state compounds. For this system, achieving agreement with experiments requires, first, an accurate electronic structure method capable of capturing the delicate hydrogen bond network governing its potential energy surface (PES),^1^ and second, sufficient sampling of the nuclear quantum motion on this PES.^2^ Generally, satisfying both criteria simultaneously is challenging, often requiring a trade-off between the accuracy of the electronic structure method and the computational efficiency for reliable sampling.

In the recent decade, the rise of machine learning potentials (MLPs) has helped to alleviate the aforementioned cost-accuracy trade-off, serving as efficient surrogate models trained to reproduce electronic structure methods.^3–6^ To date, density functional theory (DFT) has been the workhorse for generating reference training data for MLPs, owing to its mature implementation of periodic boundary conditions (PBC)—the most natural means of describing condensed phase systems—and availability of energy gradients (forces) that make it cost-effective for generating suitable datasets for training condensed phase MLPs. The utility of DFT-trained MLPs for water has been demonstrated in several recent works, enabling new insight into its condensed phase thermodynamics, particularly its phase diagram^7,8^ and how its intricate hydrogen bond network governs its unique properties.^9^ However, there are systematic and clear failings in DFT that can prevent the reliable reproduction of the experiments. For the case of liquid water, there is a common overstructuring of the radial distribution function (RDF).^1,10,11^ While combining hybrid functionals, dispersion corrections, and treatment of NQEs can improve specific structural and dynamical properties,^7,12,13^ more broadly, there have been ongoing challenges in predicting the position of the density maximum and the density ordering between ice and liquid water with DFT^11,14^ as well as the general phase diagram of water.^15,16^

To faithfully describe the PES, the method of choice is the so-called “gold-standard” coupled cluster theory with single, double, and perturbative triple excitations [CCSD­(T)]. This method has been shown to accurately reproduce experimental results for small gas phase molecules,^17^ and more recently, for surfaces^18–22^ and materials.^23,24^ However, using it to train condensed phase MLPs presents a significant challenge, due to (1) its prohibitive cost (formally scaling as *N* ^7^ with *N* electrons), (2) while PBC implementations of CCSD­(T) exist,^25^ they are not yet at a stage where they can be routinely applied to condensed phase systems, and (3) gradients of the CCSD­(T) PES are difficult to obtain. Nevertheless, recent work has highlighted promising approaches to bypass these bottlenecks for liquid water by using MLPs. Daru and co-workers used a Δ-learning strategy—previously explored for molecules^26^ where the difference between CCSD­(T) with the domain-based local pair natural orbital (DLPNO) approximation^27^ and a more affordable level of theory is fitted, showing that energies (without gradients) are sufficient to train on gas phase clusters.^28^ This Δ-MLP is added onto a baseline MLP trained to the lower-level of theory (such as DLPNO-accelerated second-order Møller–Plesset perturbation theory (MP2) and periodic DFT^29^) to reach the final desired level of accuracy. Separately, Chen et al. showed that combining new PBC implementations of CCSD­(T)—made more efficient with the frozen natural orbital approximation—with transfer learning enables data-efficient learning from small computationally tractable unit cells.^30^

The aforementioned works have highlighted the utility and promise of CCSD­(T)-level MLPs, reaching agreement with experiments across select properties of liquid water. Such agreement has also previously been achieved by alternative models based upon the many-body expansion (MBE), such as MB-pol^31^ or q-AQUA-pol,^32^ which have achieved great insight into water’s complex phase behavior.^16^ One of the medium-term goals of MLPs is to utilize their general applicability and easily derivable nature for diverse systems, while retaining the same quality realized by MBE approaches.^15,16,33^ This is crucial in order to improve over the MBE which—despite its successes—suffers from the permutational growth in body terms, steep rise in complexity with number of species, fixed topology, and bespoke development process, although there has been some recent progress in addressing these challenges.^34,35^

There are open questions that need to be addressed to enable routine CCSD­(T)-level MLPs for condensed phase simulations. First, to date, no CCSD­(T) MLP model of liquid water has been applied at constant pressure, which is important to fully resolve the isothermal–isobaric properties of a system. For liquid water, it is necessary to predict the equilibrium density of water, which exhibits a well-known (anomalous) density maximum as a function of temperature. Accurately describing density fluctuations is especially challenging when models are trained only on clusters, as missing long-range interactions from utilizing short-range MLPs tend to increase the predicted density.^36,37^ This has been explored at the DFT level by Zaverkin et al.^36^ who showed that MLPs trained directly on water clusters can predict errors in the density of up to 10% w.r.t. a model trained on periodic data. New approaches are needed that can accurately predict the density to within 1–3% from cluster data.

The previously discussed CCSD­(T) MLP models of water have heralded the start of CCSD­(T) MLPs for the condensed phase. However, they were also *tour de force* efforts, requiring significant computational investment, thereby limiting the efficient and routine development of such models. For example, the study by Daru et al. required roughly 3000 and 13,000 clusters of 64 waters at the CCSD­(T) and MP2 level, respectively, amounting to ∼3.7 million core hours to compute, while the computational cost of periodic CCSD­(T) limited Chen et al. to small 16-water molecule boxes. To lower these costs, it will be important to benchmark the effect of electronic structure parameters, namely, the basis set and the thresholds, on the local approximations to CCSD­(T)—on condensed phase properties. Such benchmarks until now have typically focused on gas phase energetics, which are difficult to connect to thermodynamic observables. This was briefly explored by Chen et al. for two basis sets (TZV2P from VandeVondele and Hutter^38^ and cc-pVQZ from Dunning^39^), which reported marked changes in the OH stretch peak. However, a systematic study, particularly involving the typical hierarchy of basis sets from double (DZ), to triple (TZ) and quadruple (QZ) ζ in size, such as within the correlation consistent (cc) basis set family, has not been performed, as well as the effect of two-point complete basis set extrapolations^40^ commonly employed to enable smaller basis sets.

In this work, we present a practical and efficient blueprint for developing MLP models at the CCSD­(T) level of theory, bringing together the many advances described above towards the accurate simulation of the thermodynamics of liquid water. We tackle the three challenges described in the previous paragraph as follows: (1) We build on the aforementioned Δ-learning strategies for CCSD­(T) MLPs, to now enable simulations at constant pressure, allowing the density properties of water to be probed, specifically the density isobar of water. (2) We present a computationally efficient approach to achieve CCSD­(T) level MLPs, enabled by improved data efficiency by using the MACE MLP approach as well as cheaper electronic structure settings, leading to over 2 orders of magnitude cheaper costs. We have validated (2) by benchmarking the (3) effect of the electronic structure parameters directly on the experimental thermodynamic properties of interest.

## 2 Reaching a Converged CCSD(T) Delta-MLP

In this section, we propose several new developments to the Δ-learning framework of Daru and co-workers.^28,29^ Our approach generates a cost-efficient dataset, enabling routine development of CCSD­(T)-level MLPs that can handle constant-pressure condensed phase simulations, which we will later leverage to predict the density of liquid water. Figure 1a summarizes the Δ-learning approach used within this work to develop CCSD­(T) MLPs for liquid water. Starting from a “baseline” MLP trained to periodic DFT data, a further Δ-MLP is fitted to elevate the PES to the CCSD­(T) level. This Δ-MLP is trained on energy differences (without gradients) between the baseline DFT and CCSD­(T) from gas phase clusters extracted from equilibrium molecular dynamics simulations. We further exploit various local CCSD­(T) approximations, such as the aforementioned DLPNO as well as the local natural orbital (LNO)^41,42^ approximation, to enable tractable calculations of much larger clusters than feasible with canonical CCSD­(T). The final CCSD­(T) MLP is the sum of (energies and forces) predicted by the baseline and Δ-MLPs, which is used to subsequently predict the structural and dynamical properties of liquid water. Figure 1b quantitatively demonstrates the reduced complexity of the fitting task for the Δ-MLP model relative to the baseline. Here we compare the distribution of predicted forces on a set of periodic snapshots by the baseline, CCSD­(T), and Δ-MLP models, where the predicted forces are on average 2 orders of magnitude lower for the Δ-MLP model.

**Figure 1:**
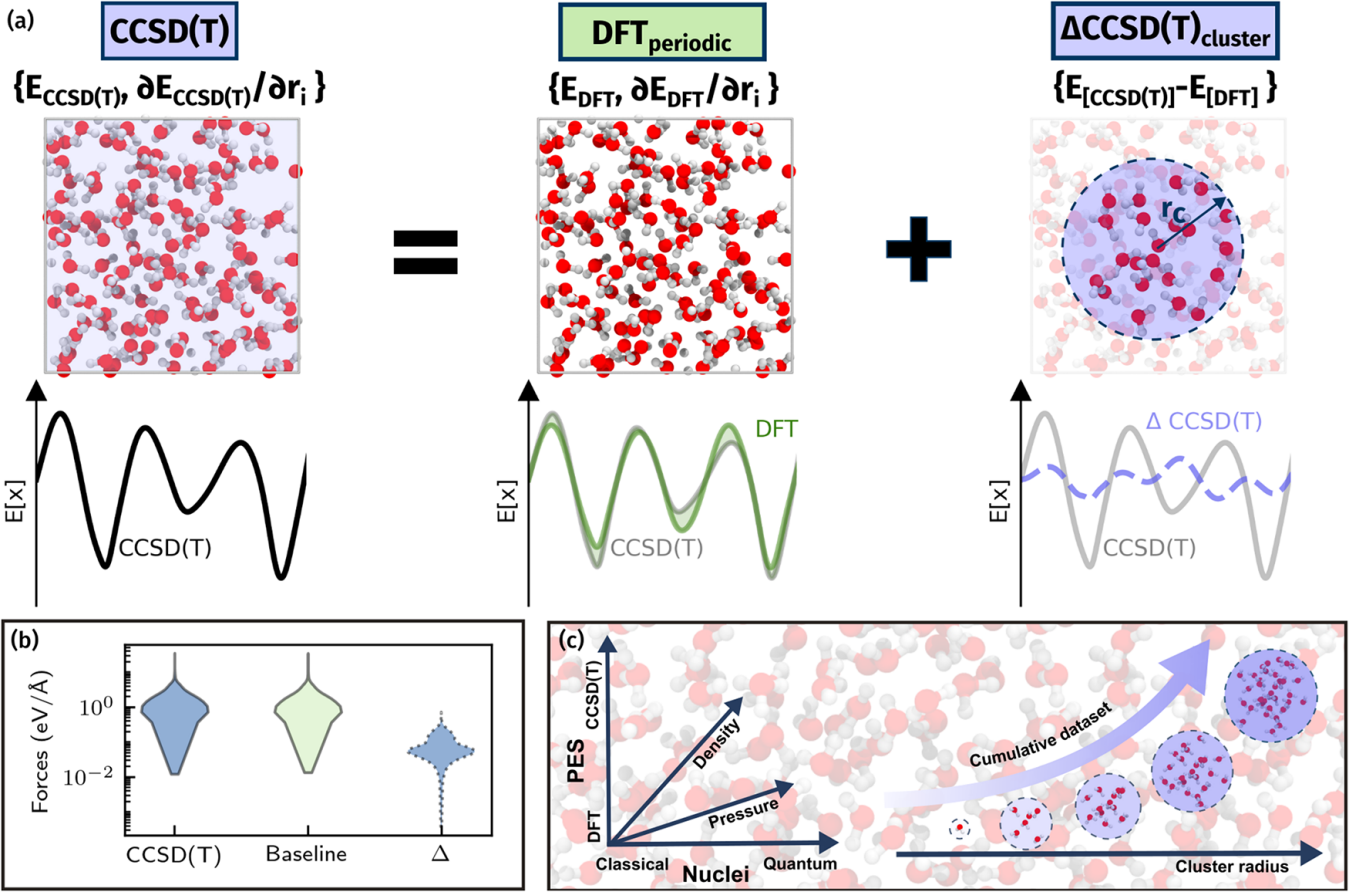
Schematic of the approach to reach CCSD­(T) accuracy for liquid water. (a) A periodic DFT MLP model is corrected via a Δ-MLP model trained on the energy difference of gas phase clusters between DFT and CCSD­(T). The relative magnitudes of the PESs to be learned are illustrated schematically below each snapshot. (b) Violin plots of the predicted force distributions of the periodic dataset by the CCSD­(T), baseline, and Δ-MLP models. (c) Schematic of the conditions sampled for generating the periodic baseline and Δ-MLP datasets. The Δ-MLP model dataset contains cumulatively increasing cluster radii.

As demonstrated by Zaverkin et al.^36^ and Kovács et al.,^37^ up until now, MLPs trained directly on clusters cannot accurately simulate under constant pressure, resulting in an overestimation of the density. A key observation of this current work is that if the baseline MLP trained to periodic DFT data can reliably perform constant-pressure simulations, the resulting CCSD­(T) MLP can yield accurate constant-pressure simulations with a Δ-MLP trained to cluster data. While the use of a baseline trained to a periodic MBE-based potential (fitted to DFT) was performed by Mészáros et al.,^29^ their work did not highlight its utility for constant-pressure simulations.

Figure 1c highlights the dataset considerations in this work in order to ensure reliable simulations under constant pressure. The baseline dataset includes configurations sampled across a wide range of thermodynamic conditions, encompassing various pressures and densities. Nuclei described both classically and including nuclear quantum effects (NQEs) via path integral molecular dynamics (PIMD) simulations are also sampled, as well as configurations from both the baseline and CCSD­(T) simulations. This diverse pool of structures is also directly used to generate the clusters used for the Δ-MLP dataset. While previous works have focused on generating datasets with clusters of a fixed number of water molecules or radius, we show in Section S6 of the SI and schematically in Figure 1c that datasets containing clusters of cumulatively increasing sizes allow for significantly fewer large clusters, which take up the significant contribution to the computational costs of evaluating the dataset.

A particular advantage of our proposed approach is that it provides a “self-consistent” means to validate the accuracy of the Δ-MLP. The cluster dataset is generated by progressively incorporating clusters of increasing sizes until the properties of interest converge. It assumes that the condensed phase properties can be learned in the limit of large cluster sizes, and we have demonstrated that the differences between two DFT levels can be learned with such an approach in Section S2.1 of the SI (where in this case we can compare to models trained on periodic data). This has also been demonstrated by Mészáros et al.^29^ between two MBE-based potentials. In Figure 2a, we plot the convergence of cumulative datasets containing cluster sizes up to a radius (*r*_c_) of 7.5 Å, going up in intervals of 1.0 Å starting from 2.5 Å, with ∼1800 structures at each cluster size. We directly compare how the resulting CCSD­(T) MLPs converge key thermodynamic observables such as the density, self-diffusion coefficient, and radial distribution function (RDF)—specifically the first peak of the O–O RDF—at ambient conditions. There is a well-controlled convergence for all three properties, with maximum cluster size, and we find that a radius of 5.5 Å—corresponding to ∼23 water molecules—is required to converge all three properties, reproducing the density to within 0.1% of the 7.5 Å result, the diffusion constant to within 4.5%, and the O–O RDF peak to within 0.01. These tests use a revPBE-D3 DFT baseline, which severely underestimates the density by almost 10% and the Δ-MLP corrects the DFT to CCSD­(T) level and close to experiment (0.997 g/cm^3^ at 298 K) while also bringing the other observables in a direction toward experiment upon inclusion of nuclear quantum effects, as will be shown in the next section.

**Figure 2:**
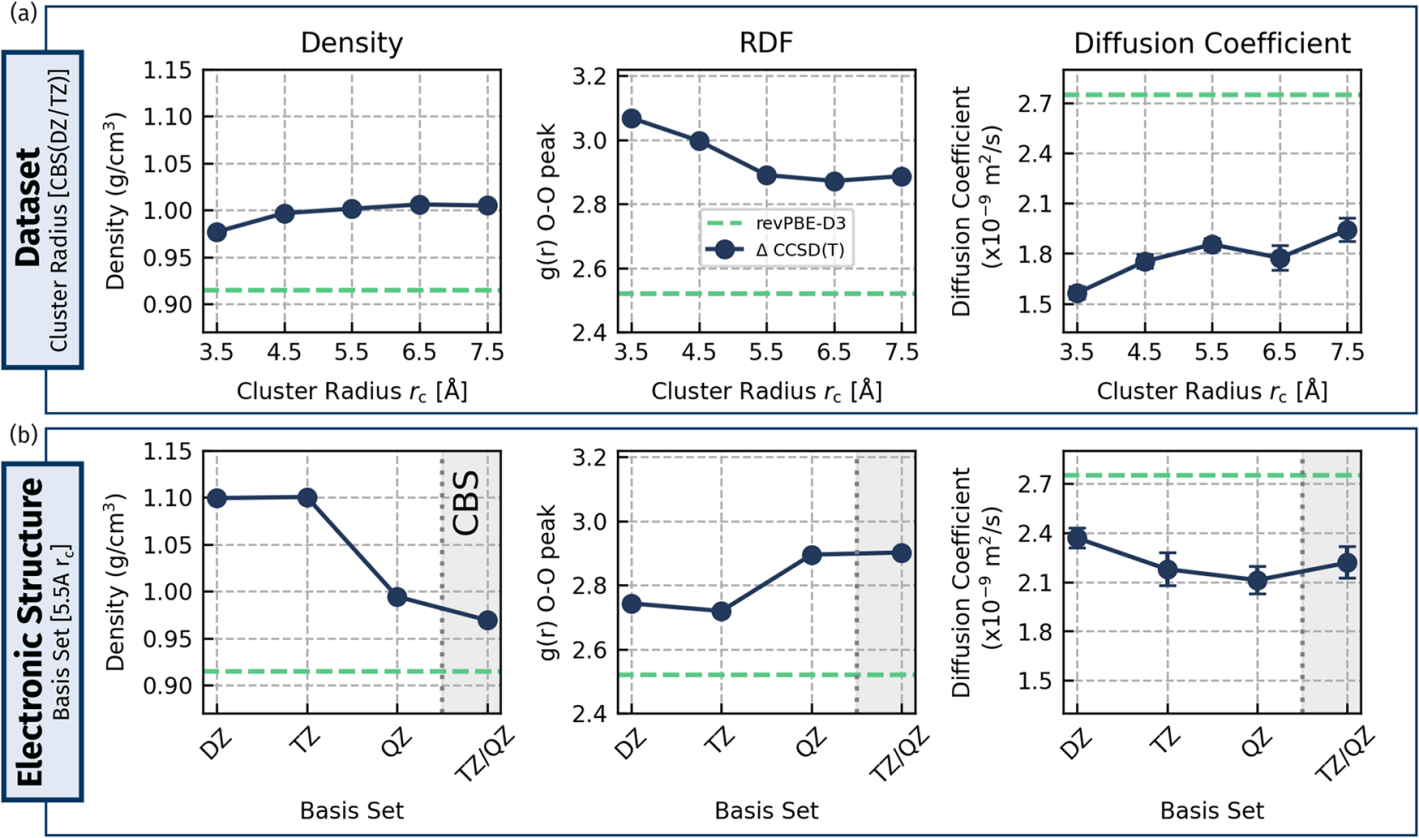
A “self-consistent” approach to determine convergence of the Δ-MLP dataset. We converge the (a) cluster size and (b) CCSD­(T) basis set for the structural and dynamical properties of bulk liquid water with classical nuclei (specifically the density, first O–O RDF peak height, and diffusion coefficient). The cluster radius datasets are a cumulative sum of all smaller radii datasets up to a given cutoff *r*_*c*_. The shaded gray region in the lower plots indicates complete basis set (CBS) extrapolation. Here, the periodic baseline is revPBE-D3, the results of which are also shown for reference with a green dashed line. The cluster size tests are done at the CBS­(DZ/TZ) level of theory, while basis set tests are done with the 5.5 Å dataset.

The dataset convergence tests were performed at the CBS­(DZ/TZ) level of theory because larger basis sets at the CCSD­(T) level for the 6.5 and 7.5 Å clusters have a prohibitively large computational cost. Regardless, we expect these observations to persist for larger basis sets given the clear convergence observed in Figure 2. Toward this end, with the converged dataset (up to 5.5 Å), we have tested basis sets increasing in size from double-ζ (DZ) to triple-ζ (TZ) and quadruple-ζ (QZ) for the jul-cc-pV*X*Z basis family,^43^ as well as the reliability of two-point extrapolation to the complete basis set (CBS) limit in Figure 2 (bottom). Our tests indicate that the QZ basis set is well converged for the thermodynamic properties studied here. The density is highly sensitive to the basis set, with a decrease of 10% going from TZ to QZ. While it was too costly to go toward larger basis sets, we can approximate these with CBS extrapolations.^44^ In particular, QZ is in close agreement with extrapolated CBS­(TZ/QZ) for all three properties, giving us confidence that it has converged, and we utilize the QZ basis set in our final models.

The final (dubbed “full”) dataset we use is at the jul-cc-pVQZ level and consists of ∼7000 structures comprising of ∼1800 clusters each that are 2.5, 3.5, 4.5, and 5.5 Å in radius. While the convergence tests shown here have been performed with revPBE-D3 (with zero damping) as the DFT baseline, any choice of DFT baseline is possible, and we show that the resulting properties are in agreement using both PBE-D3 (with zero damping) and r^2^SCAN as baselines in Section S2.2 of the SI. For our final simulations in subsequent sections, we have opted to use r^2^SCAN as the baseline model as its difference with CCSD­(T) is the lowest—corresponding to lower average predicted forces from the Δ-MLP model discussed in Section S2.2 of the SI.

## 3 Structure and Dynamics of CCSD(T) Water

Figure 3 and Table 1 compare the predictions of the final CCSD­(T) model, incorporating NQEs via path integral molecular dynamics (PIMD) simulations for dynamical and structural properties against experimental data. We achieve good agreement with experimental data for all properties, spanning both structural properties such as the RDF and density, as well as transport properties specifically the self-diffusion coefficient. We compare the O–O, O–H, and H–H RDFs, with the CCSD­(T) predictions overlapping experiments across all three properties in Figure 3. The diffusion coefficient is predicted at 298 K to be 0.22 ± 0.01 Å^2^/ps, within the error bars of experiment, and in addition, the predicted density of 0.989 ± 0.003 g/cm^3^ at 298 K is less than 1% from the predicted experimental value at 298 K of 0.997 g/cm^3^, as shown in Table 1. Given that our CCSD­(T) MLP model is able to handle constant-pressure simulations, we have computed both the RDFs and diffusion coefficient at the appropriate CCSD­(T) equilibrium density; this is particularly valuable because not all condensed phase systems have available experimental data on the density.

**Figure 3:**
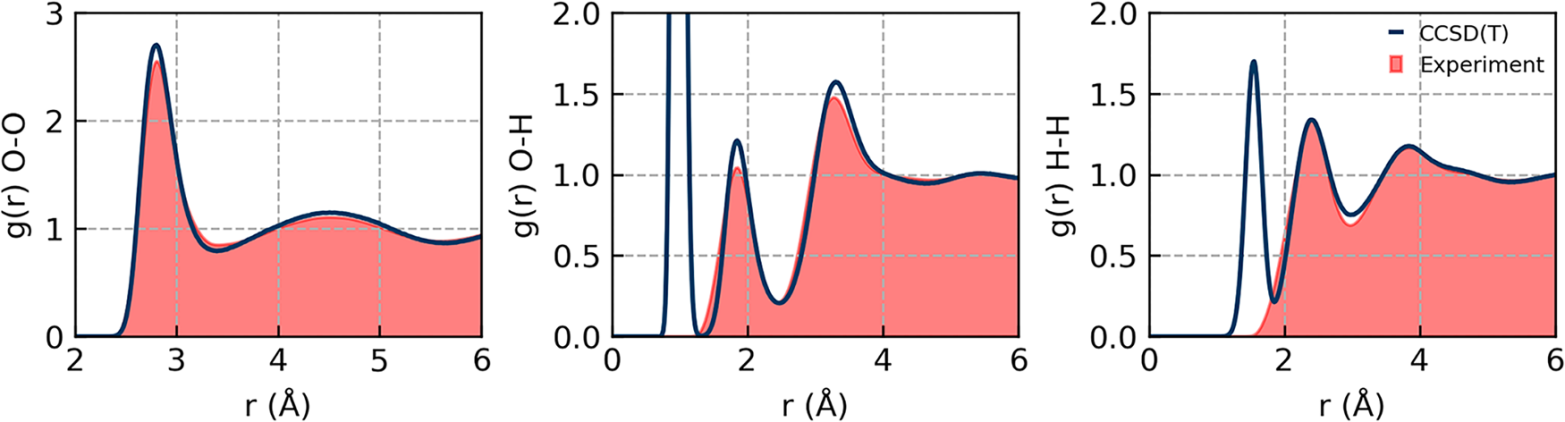
Structure of CCSD­(T) water from PIMD simulations. RDFs are computed at the equilibrium CCSD­(T) density of 0.989 g/cm^3^ that has been obtained from NPT simulations. The experimental O–O RDF data is taken from a temperature interpolation of X-ray diffraction data from ref 45 to 298 K by Daru et al.^28^ O–H and H–H RDFs are from total scattering data from ref 46.

**Table 1:** Summary of Density and Self-Diffusion Coefficient from PIMD Simulations at 298 K Compared to Experiment^a^

	ρ [g/cm^3^]	*D* [A^2^/ps]
experiment	0.997	0.23
CCSD(T)	0.989 (0.003)	0.22 (0.01)

a Simulation estimates are corrected for finite size effects and standard errors from 9 independent simulations are reported in parentheses.

The good agreement with experiment has been enabled by the combination of not only a high-quality CCSD­(T) MLP, but also the incorporation of NQEs. In Section S5.1 of the SI, we show the predictions arising from classical molecular dynamics without NQEs. We find that neglecting NQEs leads to an overstructuring of the RDFs and a lowering of the diffusion coefficient. These results are in line with previous work suggesting a roughly 1.15 increase on diffusion from inclusion of NQEs.^47^ On the other hand, the effect of NQEs on the density is negligible, with a slight decrease upon their inclusion in line with previous works.^48^

The generalizability of the models can be explored by computing the density and other properties of water beyond ambient conditions. We showcase this for the density isobar of water between 250 and 330 K in Figure 4. This is a particularly challenging property for DFT to predict, as recently shown by Montero de Hijes et al.^10^ In Figure 4, we compare our CCSD­(T) MLP predictions against a selection of common DFT functionals, taken from ref 10. With our CCSD­(T) MLP, we achieve agreement with experiments across the entire temperature range to within 1.4%. We predict a density maximum at 280 K, which is within 1.1% of the experimental value of 277 K.

**Figure 4:**
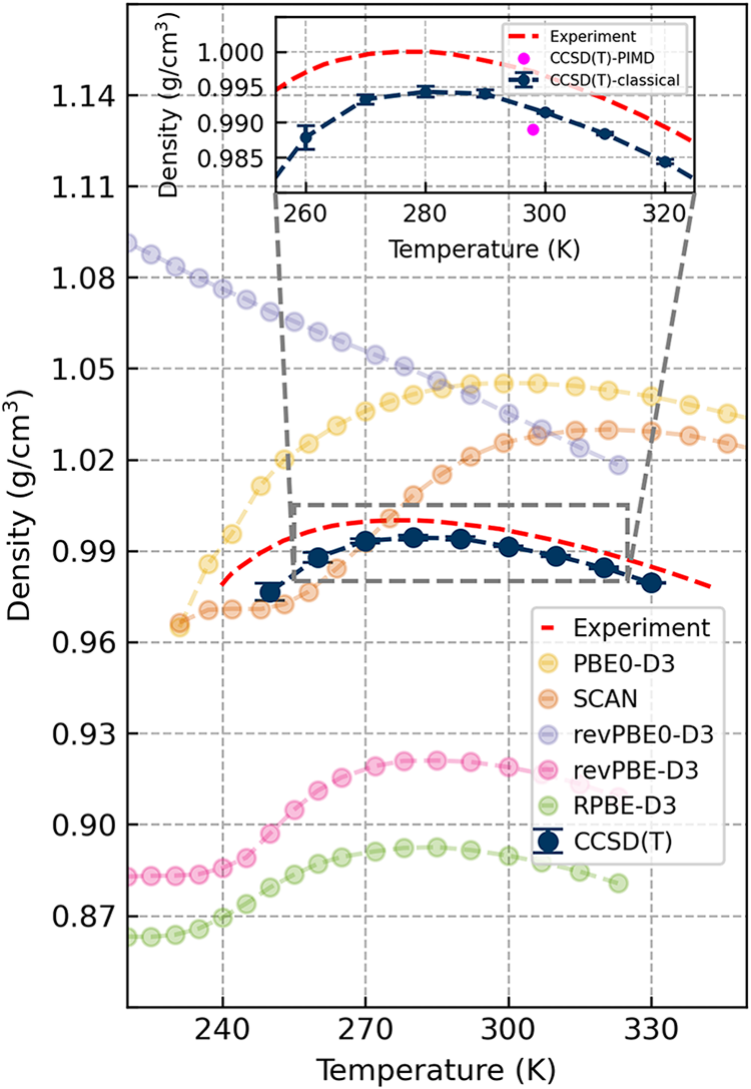
Density isobar of liquid water. The main panel compares the density isobar of the Δ CCSD­(T) model to common DFT functionals revPBE-D3, revPBE0-D3, RPBE-D3, and SCAN, as well as experiment. All simulations used classical nuclei. DFT data has been reproduced from ref 10. All D3 shown correspond to the zero damping variant of dispersion correction from Grimme et al. The inset highlights the experimental and CCSD­(T) isobars as well as showing the slight decrease in the CCSD­(T) density upon inclusion of NQEs.

## 4 Towards Cost-Effective CCSD(T) MLPs

The purpose of the above section was to accurately reach convergence of the CCSD­(T) properties without focusing on data efficiency and cost efficiency, using a large dataset as well as conservative local CCSD­(T) parameters. As a result, the dataset required roughly 1.6 million core hours to compute, as shown in Figure 5a. We show in Section S6 of the SI that the majority of this cost (87%) comes from the ∼1800 of the largest 5.5 Å radius clusters. However, we find that the number of 5.5 Å clusters can be lowered to 100 without affecting this accuracy. In particular, this was made possible because we used a cumulative dataset (incorporating clusters of smaller sizes), and we find that utilizing, e.g., only 5.5 Å clusters would require over 1500 clusters to converge all studied thermodynamic observables. This resulting dataset (containing 5500 clusters and of which only 100 are 5.5 Å in radius)—dubbed “compact dataset” achieves the same accuracy on all studied observables while being more than 1 order of magnitude cheaper. We show this for the RDFs in Figure 5c. Furthermore, without any loss in accuracy for the predictions of the thermodynamic observables, we find that the conservative “TightPNO” DLPNO approximation (∼700 core hours for each 5.5 Å cluster) can be relaxed to use the LNO approximation with “normal” thresholds, resulting in only ∼30 core hours for each 5.5 Å cluster. As shown in Section S6.3 of the SI, with all of these optimizations, the total cost of the dataset can be lowered to 15,000 core hours, requiring a maximum RAM of 50 GB, and at a cost that can be achieved within a couple of weeks on a desktop with commodity hardware. We also highlight that here the 100 clusters in the compact dataset were simply selected randomly from the full dataset of 1800 configurations. However, there is scope for more judiciously selecting these clusters using active learning type approaches such as those discussed in refs 49–51 potentially reducing the cost further. Additionally, more complex potential energy surfaces may require more targeted sampling such as those approaches proposed in ref 52.^52^

**Figure 5:**
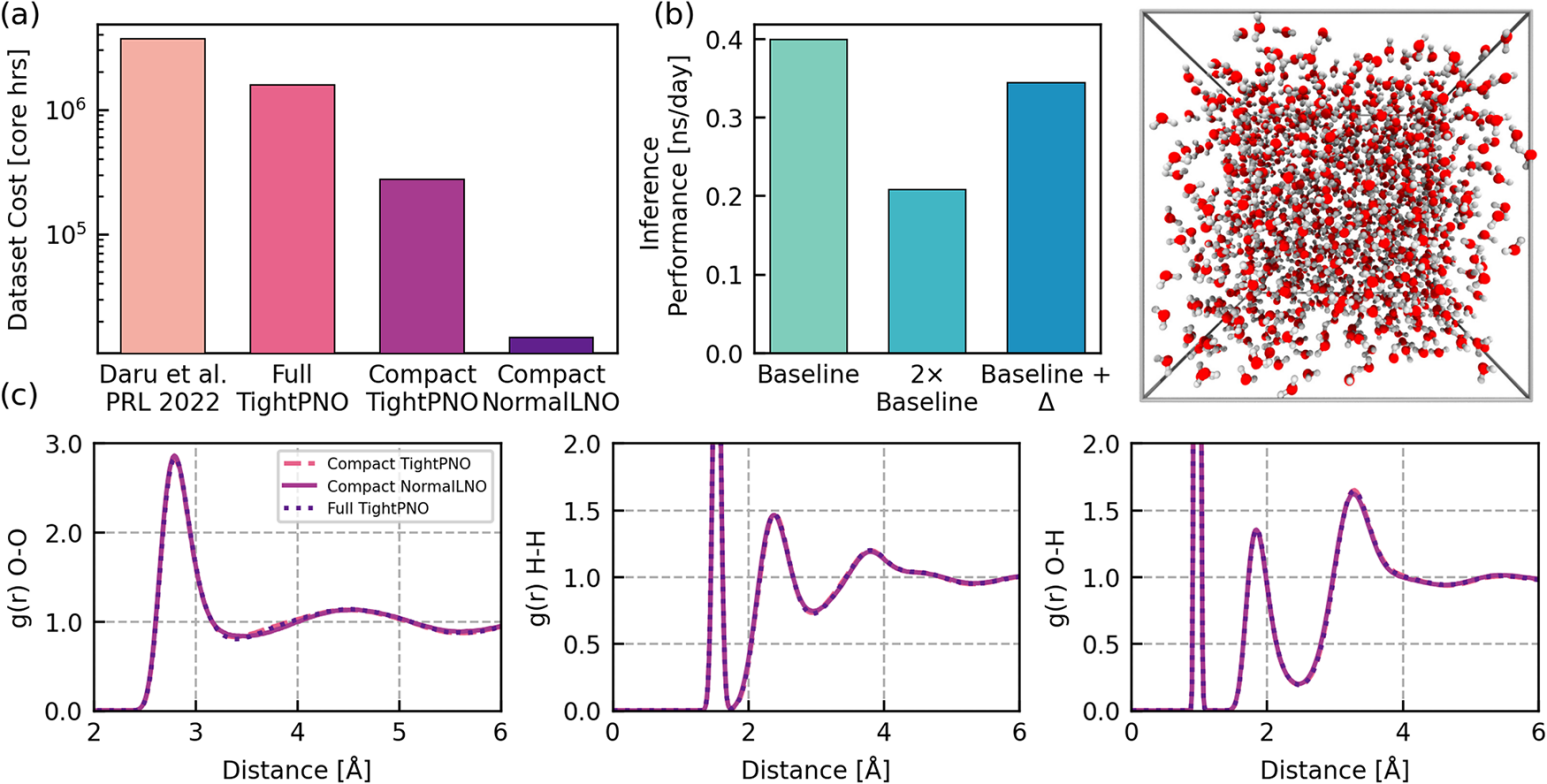
Efficient cost of the Δ-learning approach. (a) Computational cost, in number of core hours, required to generate datasets for our method compared to previous CCSD­(T) Δ-ML potential studies by Daru et al.^28^ The last three bars correspond to (i) the most conservative model using the full dataset with tight PNO thresholds in the DLPNO–CCSD­(T) approximation, (ii) the compact dataset with TightPNO thresholds in DLPNO–CCSD­(T), and (iii) the compact dataset with normal LNO thresholds (abbreviated as NormalLNO) in LNO–CCSD­(T). (b) Inference performance in ns/day (using 1 fs time step) of (1) the baseline MACE model architecture, (2) the hypothetical sum of 2 baseline architectures, and (3) the baseline and Δ-MLP model summed for a simulation box of 1000 waters shown adjacent. (c) Predicted radial distribution functions (RDFs) for the full and compact datasets and conservative and relaxed electronic structure settings shown in (a).

In principle, the disadvantage of Δ-learning is an increase in inference time (energy and gradient evaluation) due to the need to evaluate both the baseline and the Δ-MLP models. However, it has been highlighted by Mészáros et al.^29^ and Bowman et al.^53^ that the Δ-MLP can be fitted more easily than the baseline, requiring shorter cutoffs. In Section S1 of the SI, we show that a much smaller architecture compared to the baseline model is sufficient to accurately capture the bulk behavior for the Δ-MLP. Importantly, this makes the inference cost of the Δ-MLP almost negligible compared to the large MACE baseline model. Figure 5b shows only a modest reduction of approximately 10% in inference performance when summing the baseline and Δ-MLP within the final CCSD­(T) model compared to the baseline alone, significantly more efficient than the hypothetical case of summing two full baseline models. These tests were performed on a simulation box containing 1000 water molecules on 128 AMD EPYC 7742 cores, using the Symmetrix library, a C++, Kokkos optimized MACE implementation.^54–56^ Coupling the CCSD­(T) models with such efficient software further underscores the possibilities for large-scale molecular dynamics simulations with a CCSD­(T) level accuracy. As a final outlook, however, we point out that there are many properties of water, such as ice nucleation, that are still out of reach (or at the very least requiring tremendous computational cost) for these MACE MLPs (and more generally most GNN-based architectures.) Here, alternative, more efficient architectures are promising, where for example neuroevolution potentials have been shown to approach the cost of classical water potentials,^57^ as well as other architectures combining highly efficient classical descriptions with CCSD­(T) level parametrization.^58^

## 5 Discussion

The level of accuracy demonstrated in this paper for liquid water has been achieved before by previous CCSD­(T) MLP models^28,30^ as well as models based on the MBE, notably MB-pol and q-AQUA.^32,59^ Our agreement with experiment mirrors these previous works as shown in Section S5.2 of the SI. The key development in this work lies in providing a practical and generalizable approach for constructing CCSD­(T)-level MLPs for condensed phase systems. Crucially, our approach enables simulations that can be performed under constant pressure, where we demonstrate that the models can capture the density isobar of water. In particular, we have shown that it is possible to provide systematic convergence tests to identify key parameters in the training dataset that ensure resulting properties which are converged and can enable efficient development of CCSD­(T) level MLPs. The models trained in this work, datasets, and specific electronic structure inputs as well as a small code to cut the clusters are provided (see Data Availability), as a starting point for others to develop CCSD­(T) level models.

However, this current approach has some limitations that can hinder the progress towards the aforementioned goals, which we hope to address in the future. Notably, the use of a Δ-learning approach currently requires two MLP models. While we have been able to prevent the expected doubling of energy and force inference costs, it still (1) increases the labor required to develop two MLPs simultaneously, and (2) there may be an accumulation of errors from summing two models which we have not investigated in detail. Moreover, the lack of long-range electrostatics limit these models’ application to interfacial systems^60–62^ where the broken translational symmetry is not well described by purely short-ranged models.^63,64^ This (literal) shortcoming also means that they cannot simultaneously describe both clusters and the bulk, as is possible with MBE-based models. This Δ-learning approach also implicitly assumes that the DFT baseline *can* describe the long-range contributions that are missing from training purely on gas phase clusters. Learning on isolated gas phase clusters for highly charged systems may be challenging to converge with DFT methods, which would ideally require some form of embedding procedure.^65^ Solving these two problems may require higher levels of theory as a baseline such as MP2 or RPA which are much closer to the CCSD­(T) long-range behavior. Beyond Δ−learning, there are many other valid approaches toward achieving the aim of routine CCSD­(T)-quality MLPs. In particular, transfer learning and so-called multihead finetuning approaches have been shown to be particularly promising for periodic data^30,66–68^ as well as gas phase systems.^69,70^ While their efficacy in learning bulk behavior from clusters has yet to be shown (and again indeed may require explicit incorporation of long-range electrostatics), this is a promising route ready for further exploration.

With the developments outlined in this work (along with their potential limitations described above in mind), we and others are primed to tackle more challenging problems beyond the structural and dynamical properties of liquid water. We expect that it can be readily extended toward more complex aqueous solutions consisting of ions (i.e., electrolytes).^71^ We are currently applying this workflow to the ion pair association free energy of CaCO_3_ in water, where a Δ-MLP has been trained to clusters centered on the ions, with very good initial results that will be reported in a forthcoming work. Taking a further step in accurately and routinely predicting reactions occurring in various solvents would be very valuable, where high accuracy on the electronic structure front coupled with extensive sampling is necessary to reliably predict the possible reaction pathways.^72^ Finally, while this work has only focused on a limited subset of liquid water properties, there is scope towards developing a more complete water model that can tackle other challenging properties. These include the solid–liquid and liquid–air interfaces as well as describing the reactive nature of water and capturing response properties—the latter having seen recent promising progress.^73^

To summarize, we have proposed improvements to previous Δ-learning approaches to reach CCSD­(T)-quality MLPs for condensed phase simulations. Here, we summarize a blueprint as a starting point for developing and validating Δ-learned CCSD­(T) quality MLP:

1. Establish a robust NPT-capable baseline model through thorough sampling. For water, this involved sampling configuration space across a range of densities and pressures, for both classical and quantum nuclei.
2. Generate cluster datasets to train a Δ-MLP carved from bulk configurations by extracting progressively larger clusters, while retaining the smaller ones.
3. Converge observables with cluster size to identify a transferable cutoff. We find that 5.5 Å radius clusters are sufficient for liquid water, which can be used as a starting point for other systems.
4. Use reliable basis sets and local approximations as practical defaults. For liquid water, the jul-cc-pVQZ basis set along with normal/default thresholds for the LNO approximation is sufficient, serving as a starting point for future work.
5. Use efficient architectures for the Δ-correction to improve simulation performance.

Using available structural (RDF) and transport (diffusion) data of liquid water, we validated this approach, achieving good agreement with experiments. Moreover, we show that the CCSD­(T) MLP can handle constant-pressure simulations, allowing for the density isobar of water, a challenging property for DFT—to be resolved. We show our approach—based on the progressive inclusion of water clusters of increasing size—provides a systematic and straightforward means to converge and validate the dataset used to generate the Δ-MLP. This strategy enables highly data-efficient training, requiring significantly fewer large (and computationally expensive) clusters than previous methods. Finally, we perform benchmarks on the convergence of electronic structure parameters with basis set size and local approximation thresholds, as well as MLP architectures, proposing an efficient and cost-effective combination. Together, these developments enable the efficient training of CCSD­(T) MLPs, marking a further stepping stone toward routine condensed phase simulations at CCSD­(T) accuracy.

## 6 Methods

### 6.1 Machine Learning Potentials

The machine learning potentials (MLP) in this work make use of the MACE architecture,^74^ which achieves state-of-the-art accuracy and data efficiency^55^ by employing equivariant message passing with local body-order descriptions of each atom. We used a different choice of hyperparameters for the baseline and Δ-MLPs. The baseline DFT MLPs comprise 2 message passing layers with 128 channels and a 6 Å cutoff, while this is reduced to 64 channels and a 4 Å cutoff for the Δ-MLP. We show in Section S1.4 of the SI and the Discussion that these reduced settings significantly decrease the inference cost of the Δ-MLP with no compromise in accuracy. For each dataset, 10% was held back as a validation set.

### 6.2 Density Functional Theory

The DFT calculations required for both the baseline MLP and the Δ-MLP were performed with FHI-AIMs.^75^ It can perform calculations under both periodic and open boundary conditions, allowing consistent treatment of the electronic structure of both periodic and cluster calculations in the baseline and Δ-MLP, respectively. We used the tight family of basis sets and the (in-built) Grimme’s D3 ^76^ dispersion program in FHI-AIMs. The periodic unit cell calculations were performed by using the Γ-point. As described in Section S1 of the SI, the dataset used to generate our periodic and Δ-MLP models in Section S1 of the SI, consisting of ∼1000 structures in the former and ∼7000 structures in the latter, was all sampled from data generated at a wide range of pressures (with nuclear quantum effects). This dataset was computed with revPBE-D3 ^77^ (with zero damping), PBE-D3 ^78^ (with zero damping), and r^2^SCAN^79^ as the baseline.

### 6.3 Coupled Cluster Theory

The dataset of CCSD­(T) energies was calculated in both ORCA^80^ and MRCC,^81^ utilizing the domain-based local pair natural orbital (DLPNO)^27,82,83^ and local natural orbital (LNO) approximations,^41,42^ respectively. We have predominantly used the ORCA code, using conservative electronic structure parameters, namely “TightPNO” DLPNO thresholds, with resolution-of-identity approximations disabled for the initial Hartree–Fock (HF) calculations. With the MRCC code, we have aimed to go towards more cost-efficient electronic structure parameters, using the default “normal” LNO thresholds and enabling density-fitting to speed up the HF calculations. For both codes, we used the jul-cc-pV*X*Z basis sets,^43^ consisting of the aug-cc-pV*X*Z basis set on the O and cc-pV*X*Z basis set on the H atoms, together with their corresponding density-fitting basis sets in the local CCSD­(T) correlation treatment. We performed complete basis set (CBS) extrapolations for the triple (TZ) and quadruple-ζ (QZ) basis sets, using parameters taken from Neese and Valeev.^44^

### 6.4 Molecular Dynamics

With the resulting CCSD­(T) MLP, molecular dynamics simulations were performed to obtain the following observables: density isobar (and the density at 298 K and 1 bar pressure), radial distribution function (RDF), and self-diffusion coefficient. All classical simulations were performed using the Large-Scale Atomic/Molecular Massively Parallel Simulator (LAMMPS)^84^ code, coupled with the Symmetrix library,^54–56^ using the symmetrix/mace pair style in tandem with the hybrid/overlay pair style to sum the periodic and Δ-MLPs. All simulation boxes contained 126 waters and were performed at 298 K. Classical simulations used a 0.5 fs timestep. The density was obtained from simulations in the isothermal isobaric ensemble (NPT) at a pressure of 1 bar with a barostat relaxation time of 1 ps. All density simulations were run for at least 500 ps with block averaging to obtain the error bar. RDFs and self-diffusion coefficients were obtained from simulations in the canonical (NVT) ensemble. For all convergence tests, these were computed at the experimental density with a box size of 15.577 Å to ensure consistency. For the final model production simulations, the RDF and self-diffusion coefficient were obtained from simulations at the computed density from the NPT simulations. In all cases, the CSVR thermostat^85^ was used, with a temperature of 298 K and a temperature relaxation time of 0.1 ps. Path integral molecular dynamics simulations were performed using the i-PI code^86^ interfaced with LAMMPS^84^ and symmetrix.^54–56^ Densities were obtained from ring polymer molecular dynamics (RPMD)^87^ simulations in the isobaric isothermal ensemble using 32 beads, with a 0.25 fs timestep, with each replica at least 200 ps long. RDFs and diffusion coefficients were computed in the canonical ensemble using thermostatted RPMD (T-RPMD)^88^ at 298 K with a 0.25 fs timestep, using the computed density. An average of 9 trajectories of 100 ps each were taken for the final diffusion coefficient, obtained from the mean squared displacement of the centroid of the ring polymer.

## Supplementary Material



## Data Availability

The data required to reproduce this study is provided in the following Github repository: https://github.com/fast-group-cam/data_cc_water. All simulations were performed with publicly available simulation software (ACEsuit, LAMMPS, Symmetrix).

## Notes

Machine learning potentials: data set construction, Δ-learning strategy, validation and seed dependence, architecture/hyperparameters; overall validation: DFT to DFT (revPBE-D3, r2SCAN, PBE-D3) and DFT to CCSD­(T) validation; electronic structure details: CCSD­(T) protocols, basis sets/CBS extrapolations, validation of local approximations; molecular-dynamics details: classical MD, PIMD, diffusion coefficients with finite size correction, density isobar; additional results: classical CCSD­(T) RDFs, comparison to literature models (Daru et al., Chen et al., MB-pol, q-AQUA-pol); and data efficiency: convergence of training data, cost reduction via MRCC LNO, compact data set design, and memory requirements (PDF)

## References

[1] Gillan M. J., Alfè D., Michaelides A. (2016). Perspective: How Good Is DFT for
Water?. J. Chem. Phys..

[2] Ceriotti M., Fang W., Kusalik P. G., McKenzie R. H., Michaelides A., Morales M. A., Markland T. E. (2016). Nuclear Quantum Effects in Water
and Aqueous Systems: Experiment, Theory, and Current Challenges. Chem. Rev..

[3] Behler J. (2016). Perspective:
Machine Learning Potentials for Atomistic Simulations. J. Chem. Phys..

[4] Bartók A. P., De S., Poelking C., Bernstein N., Kermode J. R., Csányi G., Ceriotti M. (2017). Machine Learning Unifies the Modeling of Materials
and Molecules. Sci. Adv..

[5] Thiemann F. L., O’Neill N., Kapil V., Michaelides A., Schran C. (2025). Introduction to Machine Learning Potentials for Atomistic
Simulations. J. Phys.: Condens. Matter.

[6] Kang P.-L., Shang C., Liu Z.-P. (2020). Large-Scale
Atomic Simulation via
Machine Learning Potentials Constructed by Global Potential Energy
Surface Exploration. Acc. Chem. Res..

[7] Reinhardt A., Cheng B. (2021). Quantum-Mechanical
Exploration of the Phase Diagram of Water. Nat.
Commun..

[8] Zhang L., Wang H., Car R., Weinan E. (2021). Phase Diagram of a
Deep Potential Water Model. Phys. Rev. Lett..

[9] Morawietz T., Singraber A., Dellago C., Behler J. (2016). How van Der Waals Interactions
Determine the Unique Properties of Water. Proc.
Natl. Acad. Sci. U.S.A..

[10] Montero
de Hijes P., Dellago C., Jinnouchi R., Kresse G. (2024). Density Isobar of Water and Melting Temperature of
Ice: Assessing Common Density Functionals. J.
Chem. Phys..

[11] Chen M., Ko H.-Y., Remsing R. C., Calegari Andrade M. F., Santra B., Sun Z., Selloni A., Car R., Klein M. L., Perdew J. P., Wu X. (2017). Ab Initio Theory and
Modeling of Water. Proc. Natl. Acad. Sci. U.S.A..

[12] Marsalek O., Markland T. E. (2017). Quantum Dynamics
and Spectroscopy of Ab Initio Liquid
Water: The Interplay of Nuclear and Electronic Quantum Effects. J. Phys. Chem. Lett..

[13] Ruiz
Pestana L., Marsalek O., Markland T. E., Head-Gordon T. (2018). The Quest
for Accurate Liquid Water Properties from First Principles. J. Phys. Chem. Lett..

[14] Gaiduk A. P., Gygi F., Galli G. (2015). Density and Compressibility
of Liquid
Water and Ice from First-Principles Simulations with Hybrid Functionals. J. Phys. Chem. Lett..

[15] Bore S. L., Paesani F. (2023). Realistic Phase Diagram of Water
from “First
Principles” Data-Driven Quantum Simulations. Nat. Commun..

[16] Sciortino F., Zhai Y., Bore S. L., Paesani F. (2025). Constraints on the
Location of the Liquid-Liquid Critical Point in Water. Nat. Phys..

[17] Karton A., Rabinovich E., Martin J. M. L., Ruscic B. (2006). W4 Theory
for Computational
Thermochemistry: In Pursuit of Confident Sub-kJ/Mol Predictions. J. Chem. Phys..

[18] Shi B. X., Zen A., Kapil V., Nagy P. R., Grüneis A., Michaelides A. (2023). Many-Body
Methods for Surface Chemistry Come of Age:
Achieving Consensus with Experiments. J. Am.
Chem. Soc..

[19] Shi B. X., Rosen A. S., Schäfer T., Grüneis A., Kapil V., Zen A., Michaelides A. (2025). An Accurate
and Efficient Framework for Modelling the Surface Chemistry of Ionic
Materials. Nat. Chem..

[20] Schäfer T., Libisch F., Kresse G., Grüneis A. (2021). Local Embedding
of Coupled Cluster Theory into the Random Phase Approximation Using
Plane Waves. J. Chem. Phys..

[21] Carbone J. P., Irmler A., Gallo A., Schäfer T., Benschoten W. Z. V., Shepherd J. J., Grüneis A. (2024). CO Adsorption
on Pt(111) Studied by Periodic Coupled Cluster Theory. Faraday Discuss..

[22] Ye H.-Z., Berkelbach T. C. (2024). Adsorption
and Vibrational Spectroscopy of CO on the
Surface of MgO from Periodic Local Coupled-Cluster Theory. Faraday Discuss..

[23] Ye, H.-Z. ; Berkelbach, T. C. Periodic Local Coupled-Cluster Theory for Insulators and Metals 2024 20 8948 8959 10.1021/acs.jctc.4c00936. 39376105

[24] Yang J., Hu W., Usvyat D., Matthews D., Schütz M., Chan G. K.-L. (2014). Ab Initio Determination of the Crystalline Benzene
Lattice Energy to Sub-Kilojoule/Mole Accuracy. Science.

[25] McClain J., Sun Q., Chan G. K.-L., Berkelbach T. C. (2017). Gaussian-Based Coupled-Cluster Theory
for the Ground-State and Band Structure of Solids. J. Chem. Theory Comput..

[26] Ramakrishnan R., Dral P. O., Rupp M., von Lilienfeld O. A. (2015). Big Data
Meets Quantum Chemistry Approximations: The Δ-Machine Learning
Approach. J. Chem. Theory Comput..

[27] Riplinger C., Neese F. (2013). An Efficient and near
Linear Scaling Pair Natural Orbital Based Local
Coupled Cluster Method. J. Chem. Phys..

[28] Daru J., Forbert H., Behler J., Marx D. (2022). Coupled Cluster Molecular
Dynamics of Condensed Phase Systems Enabled by Machine Learning Potentials:
Liquid Water Benchmark. Phys. Rev. Lett..

[29] Mészáros B. B., Szabó A., Daru J. (2025). Short-Range Δ-Machine Learning:
A Cost-Efficient Strategy to Transfer Chemical Accuracy to Condensed
Phase Systems. J. Chem. Theory Comput..

[30] Chen M. S., Lee J., Ye H.-Z., Berkelbach T. C., Reichman D. R., Markland T. E. (2023). Data-Efficient
Machine Learning Potentials from Transfer Learning of Periodic Correlated
Electronic Structure Methods: Liquid Water at AFQMC, CCSD, and CCSD­(T)
Accuracy. J. Chem. Theory Comput..

[31] Palos E., Bull-Vulpe E. F., Zhu X., Agnew H., Gupta S., Saha S., Paesani F. (2024). Current Status
of the MB-pol Data-Driven
Many-Body Potential for Predictive Simulations of Water Across Different
Phases. J. Chem. Theory Comput..

[32] Qu C., Yu Q., Houston P. L., Conte R., Nandi A., Bowman J. M. (2023). Interfacing
Q-AQUA with a Polarizable Force Field: The Best of Both Worlds. J. Chem. Theory Comput..

[33] Bowman J. M., Qu C., Conte R., Nandi A., Houston P. L., Yu Q. (2025). A Perspective
Marking 20 Years of Using Permutationally Invariant Polynomials for
Molecular Potentials. J. Chem. Phys..

[34] Zhou R., Bull-Vulpe E. F., Pan Y., Paesani F. (2025). Toward Chemical Accuracy
in Biomolecular Simulations through Data-Driven Many-Body Potentials:
I. Polyalanine in the Gas Phase. J. Chem. Theory
Comput..

[35] Zhou, R. ; Paesani, F. Toward Chemical Accuracy in Biomolecular Simulations through Data-Driven Many-Body Potentials: II. Polyalanine in Water 2025 21 10574 10587 10.1021/acs.jctc.5c01335. 41104727

[36] Zaverkin V., Holzmüller D., Schuldt R., Kästner J. (2022). Predicting
Properties of Periodic Systems from Cluster Data: A Case Study of
Liquid Water. J. Chem. Phys..

[37] Kovács D. P., Moore J. H., Browning N. J., Batatia I., Horton J. T., Pu Y., Kapil V., Witt W. C., Magdău I.-B., Cole D. J., Csányi G. (2025). MACE-OFF: Short-Range Transferable
Machine Learning Force Fields for Organic Molecules. J. Am. Chem. Soc..

[38] VandeVondele J., Hutter J. (2007). Gaussian Basis Sets for Accurate Calculations on Molecular
Systems in Gas and Condensed Phases. J. Chem.
Phys..

[39] Peterson K. A., Dunning T. H. (2002). Accurate Correlation Consistent Basis Sets for Molecular
Core-Valence Correlation Effects: The Second Row Atoms Al-Ar, and
the First Row Atoms B-Ne Revisited. J. Chem.
Phys..

[40] Feller D., Peterson K. A., Grant
Hill J. (2011). On the Effectiveness of CCSD­(T) Complete
Basis Set Extrapolations for Atomization Energies. J. Chem. Phys..

[41] Nagy P. R., Samu G., Kállay M. (2018). Optimization
of the Linear-Scaling
Local Natural Orbital CCSD­(T) Method: Improved Algorithm and Benchmark
Applications. J. Chem. Theory Comput..

[42] Gyevi-Nagy L., Kállay M., Nagy P. R. (2020). Integral-Direct and Parallel Implementation
of the CCSD­(T) Method: Algorithmic Developments and Large-Scale Applications. J. Chem. Theory Comput..

[43] Papajak E., Zheng J., Xu X., Leverentz H. R., Truhlar D. G. (2011). Perspectives on Basis Sets Beautiful: Seasonal Plantings
of Diffuse Basis Functions. J. Chem. Theory
Comput..

[44] Neese F., Valeev E. F. (2011). Revisiting the Atomic
Natural Orbital Approach for
Basis Sets: Robust Systematic Basis Sets for Explicitly Correlated
and Conventional Correlated *Ab Initio Methods?*. J. Chem. Theory Comput..

[45] Skinner L. B., Benmore C. J., Neuefeind J. C., Parise J. B. (2014). The Structure of
Water around the Compressibility Minimum. J.
Chem. Phys..

[46] Soper A. K. (2013). The Radial
Distribution Functions of Water as Derived from Radiation Total Scattering
Experiments: Is There Anything We Can Say for Sure?. Int. Scholarly Res. Not..

[47] Habershon S., Markland T. E., Manolopoulos D. E. (2009). Competing Quantum Effects in the
Dynamics of a Flexible Water Model. J. Chem.
Phys..

[48] Medders G. R., Babin V., Paesani F. (2014). Development of a “First-Principles”
Water Potential with Flexible Monomers. III. Liquid Phase Properties. J. Chem. Theory Comput..

[49] Käser S., Richardson J. O., Meuwly M. (2025). Transfer Learning for Predictive
Molecular Simulations: Data-Efficient Potential Energy Surfaces at
CCSD­(T) Accuracy. J. Chem. Theory Comput..

[50] Käser S., Richardson J. O., Meuwly M. (2022). Transfer Learning for Affordable
and High-Quality Tunneling Splittings from Instanton Calculations. J. Chem. Theory Comput..

[51] Schran C., Thiemann F. L., Rowe P., Müller E. A., Marsalek O., Michaelides A. (2021). Machine Learning Potentials for Complex
Aqueous Systems Made Simple. Proc. Natl. Acad.
Sci. U.S.A..

[52] Zhang H., Juraskova V., Duarte F. (2024). Modelling Chemical Processes in Explicit
Solvents with Machine Learning Potentials. Nat.
Commun..

[53] Bowman J. M., Qu C., Conte R., Nandi A., Houston P. L., Yu Q. (2023). Δ-Machine
Learned Potential Energy Surfaces and Force Fields. J. Chem. Theory Comput..

[54] Witt, W. C. Symmetrix, Available at: https://github.com/wcwitt/symmetrix. 2025.

[55] Kovács D. P., Batatia I., Arany E. S., Csányi G. (2023). Evaluation
of the MACE Force Field Architecture: From Medicinal Chemistry to
Materials Science. J. Chem. Phys..

[56] Batatia I., Benner P., Chiang Y., Elena A. M., Kovács D. P., Riebesell J., Advincula X. R., Asta M., Avaylon M., Baldwin W. J., Berger F., Bernstein N., Bhowmik A., Bigi F., Blau S. M., Cărare V., Ceriotti M., Chong S., Darby J. P., De S., Pia F. D., Deringer V. L., Elijošius R., El-Machachi Z., Falcioni F., Fako E., Ferrari A. C., Gardner J. L. A., Gawkowski M. J., Genreith-Schriever A., George J., Goodall R. E. A., Grandel J., Grey C. P., Grigorev P., Han S., Handley W., Heenen H. H., Hermansson K., Holm C., Ho C. H., Hofmann S., Jaafar J., Jakob K. S., Jung H., Kapil V., Kaplan A. D., Karimitari N., Kermode J. R., Kourtis P., Kroupa N., Kullgren J., Kuner M. C., Kuryla D., Liepuoniute G., Lin C., Margraf J. T., Magdău I.-B., Michaelides A., Moore J. H., Naik A. A., Niblett S. P., Norwood S. W., O’Neill N., Ortner C., Persson K. A., Reuter K., Rosen A. S., Rosset L. A. M., Schaaf L. L., Schran C., Shi B. X., Sivonxay E., Stenczel T. K., Svahn V., Sutton C., Swinburne T. D., Tilly J., van der Oord C., Vargas S., Varga-Umbrich E., Vegge T., Vondrák M., Wang Y., Witt W. C., Wolf T., Zills F., Csányi G. (2025). A Foundation
Model for Atomistic Materials Chemistry. arXiv.

[57] Xu K., Liang T., Xu N., Ying P., Chen S., Wei N., Xu J., Fan Z. (2025). NEP-MB-pol: A Unified Machine-Learned
Framework for Fast and Accurate Prediction of Water’s Thermodynamic
and Transport Properties. npj Comput. Mater..

[58] Boittier E. D., Käser S., Meuwly M. (2025). Roadmap to CCSD­(T)-Quality Machine-Learned
Potentials for Condensed Phase Simulations. J. Chem. Theory Comput..

[59] Yu Q., Qu C., Houston P. L., Nandi A., Pandey P., Conte R., Bowman J. M. (2023). A Status Report on “Gold Standard”
Machine-Learned
Potentials for Water. J. Phys. Chem. Lett..

[60] Fitzner M., Sosso G. C., Cox S. J., Michaelides A. (2015). The Many Faces
of Heterogeneous Ice Nucleation: Interplay Between Surface Morphology
and Hydrophobicity. J. Am. Chem. Soc..

[61] Finney A. R., Salvalaglio M. (2022). Multiple Pathways
in NaCl Homogeneous Crystal Nucleation. Faraday
Discuss..

[62] O’Neill N., Schran C., Cox S. J., Michaelides A. (2024). Crumbling
Crystals: On the Dissolution Mechanism of NaCl in Water. Phys. Chem. Chem. Phys..

[63] Niblett S. P., Galib M., Limmer D. T. (2021). Learning Intermolecular Forces at
Liquid-Vapor Interfaces. J. Chem. Phys..

[64] Yue S., Muniz M. C., Calegari Andrade M. F., Zhang L., Car R., Panagiotopoulos A. Z. (2021). When Do Short-Range Atomistic Machine-Learning Models
Fall Short?. J. Chem. Phys..

[65] Huang C., Pavone M., Carter E. A. (2011). Quantum Mechanical Embedding Theory
Based on a Unique Embedding Potential. J. Chem.
Phys..

[66] Kaur H., Pia F. D., Batatia I., R Advincula X., X Shi B., Lan J., Csányi G., Michaelides A., Kapil V. (2025). Data-Efficient Fine-Tuning of Foundational
Models for First-Principles Quality Sublimation Enthalpies. Faraday Discuss..

[67] Gawkowski, M. J. ; Li, M. ; Shi, B. X. ; Kapil, V. The Good, the Bad, and the Ugly of Atomistic Learning for “Clusters-to-Bulk” Generalization. arXiv:2509.16601. arXiv.org e-Print archive. https://arxiv.org/abs/2509.16601. 2025.

[68] Cui M., Reuter K., Margraf J. T. (2025). Multi-Fidelity
Transfer Learning
for Quantum Chemical Data Using a Robust Density Functional Tight
Binding Baseline. Mach. Learn.: Sci. Technol..

[69] Käser S., Meuwly M. (2023). Transfer-Learned Potential
Energy Surfaces: Toward
Microsecond-Scale Molecular Dynamics Simulations in the Gas Phase
at CCSD­(T) Quality. J. Chem. Phys..

[70] Messerly M., Matin S., Allen A. E. A., Nebgen B., Barros K., Smith J. S., Lubbers N., Messerly R. (2025). Multi-Fidelity Learning
for Interatomic Potentials: Low-level Forces and High-Level Energies
Are All You Need. Mach. Learn.: Sci. Technol..

[71] O’Neill N., Shi B. X., Fong K., Michaelides A., Schran C. (2024). To Pair or Not to Pair? Machine-Learned Explicitly-Correlated
Electronic Structure for NaCl in Water. J. Phys.
Chem. Lett..

[72] Young T. A., Johnston-Wood T., L Deringer V., Duarte F. (2021). A Transferable Active-Learning
Strategy for Reactive Molecular Force Fields. Chem. Sci..

[73] Jindal A., Schienbein P., Das B., Marx D. (2025). Computing Bulk Phase
IR Spectra from Finite Cluster Data via Equivariant Neural Networks. J. Chem. Theory Comput..

[74] Batatia, I. ; Kovacs, D. P. ; Simm, G. ; Ortner, C. ; Csanyi, G. MACE: Higher Order Equivariant Message Passing Neural Networks for Fast and Accurate Force Fields. In Advances in Neural Information Processing Systems. 2022; pp 11423–11436.

[75] Blum V., Gehrke R., Hanke F., Havu P., Havu V., Ren X., Reuter K., Scheffler M. (2009). *Ab Initio* Molecular
Simulations with Numeric Atom-Centered Orbitals. Comput. Phys. Commun..

[76] Grimme S., Antony J., Ehrlich S., Krieg H. (2010). A Consistent
and Accurate
Ab Initio Parametrization of Density Functional Dispersion Correction
(DFT-D) for the 94 Elements H-Pu. J. Chem. Phys..

[77] Zhang Y., Yang W. (1998). Comment on “Generalized Gradient Approximation Made Simple. Phys. Rev. Lett..

[78] Perdew J. P., Burke K., Ernzerhof M. (1996). Generalized Gradient Approximation
Made Simple. Phys. Rev. Lett..

[79] Furness J. W., Kaplan A. D., Ning J., Perdew J. P., Sun J. (2020). Accurate and
Numerically Efficient r^2^SCAN Meta-Generalized Gradient
Approximation. J. Phys. Chem. Lett..

[80] Neese F., Wennmohs F., Becker U., Riplinger C. (2020). The ORCA Quantum
Chemistry Program Package. J. Chem. Phys..

[81] Kállay M., Nagy P. R., Mester D., Rolik Z., Samu G., Csontos J., Csóka J., Szabó P. B., Gyevi-Nagy L., Hégely B., Ladjánszki I., Szegedy L., Ladóczki B., Petrov K., Farkas M., Mezei P. D., Ganyecz Á. (2020). The MRCC Program System: Accurate
Quantum Chemistry from Water to Proteins. J.
Chem. Phys..

[82] Riplinger C., Sandhoefer B., Hansen A., Neese F. (2013). Natural Triple Excitations
in Local Coupled Cluster Calculations with Pair Natural Orbitals. J. Chem. Phys..

[83] Riplinger C., Pinski P., Becker U., Valeev E. F., Neese F. (2016). Sparse Maps–A
Systematic Infrastructure for Reduced-Scaling Electronic Structure
Methods. II. Linear Scaling Domain Based Pair Natural Orbital Coupled
Cluster Theory. J. Chem. Phys..

[84] Thompson A. P., Aktulga H. M., Berger R., Bolintineanu D. S., Brown W. M., Crozier P. S., in ’t Veld P. J., Kohlmeyer A., Moore S. G., Nguyen T. D., Shan R., Stevens M. J., Tranchida J., Trott C., Plimpton S. J. (2022). LAMMPS
- a Flexible Simulation Tool for Particle-Based Materials Modeling
at the Atomic, Meso, and Continuum Scales. Comput.
Phys. Commun..

[85] Bussi G., Donadio D., Parrinello M. (2007). Canonical
Sampling through Velocity
Rescaling. J. Chem. Phys..

[86] Litman Y., Kapil V., Feldman Y. M. Y., Tisi D., Begušić T., Fidanyan K., Fraux G., Higer J., Kellner M., Li T. E., Pós E. S., Stocco E., Trenins G., Hirshberg B., Rossi M., Ceriotti M. (2024). I-PI 3.0: A Flexible
and Efficient Framework for Advanced Atomistic Simulations. J. Chem. Phys..

[87] Craig I. R., Manolopoulos D. E. (2004). Quantum
Statistics and Classical Mechanics: Real Time
Correlation Functions from Ring Polymer Molecular Dynamics. J. Chem. Phys..

[88] Rossi M., Ceriotti M., Manolopoulos D. E. (2014). How to Remove the Spurious Resonances
from Ring Polymer Molecular Dynamics. J. Chem.
Phys..

