# Supplementary material for: Towards Routine Condensed Phase Simulations with Delta-Learned Coupled Cluster Accuracy: Application to Liquid Water
Source: J Chem Theory Comput. 2025 Nov 7;21(22):11710–20. doi: 10.1021/acs.jctc.5c01377 (PMC12659020; doi:10.1021/acs.jctc.5c01377)
Supplement: Supplementary file 1 [file ct5c01377_si_001.pdf]

# Supporting Information for: "Towards Routine Condensed Phase Simulations with Delta-Learned Coupled Cluster Accuracy: Application to Liquid Water"

Niamh O'Neill,[*,†,‡,¶] Benjamin X. Shi,[*,§] William J. Baldwin,[¶,‖] William C. Witt,[⊥] Gábor Csányi,[¶,‖] Julian D. Gale,[#] Angelos Michaelides,[†,¶] and Christoph Schran[*,‡,¶]

†*Yusuf Hamied Department of Chemistry, University of Cambridge, Lensfield Road, Cambridge, CB2 1EW, UK*

‡*Cavendish Laboratory, Department of Physics, University of Cambridge, Cambridge, CB3 0HE, UK*

¶*Lennard-Jones Centre, University of Cambridge, Trinity Ln, Cambridge, CB2 1TN, UK*

§*Initiative for Computational Catalysis, Flatiron Institute, 160 5th Avenue, New York, NY 10010*

‖*Department of Engineering, University of Cambridge, Cambridge, CB3 0HE, UK*

⊥*Harvard John A. Paulson School of Engineering and Applied Sciences, Harvard University, Cambridge, MA, USA*

#*School of Molecular and Life Sciences, Curtin University, PO Box U1987, Perth, Western Australia 6845, Australia*

E-mail: nco24@cam.ac.uk; mail@benjaminshi.com; cs2121@cam.ac.uk

# Contents


**S1 Machine learning** **3**

S1.1 Dataset . . . . . . . . . . . . . . . . . . . . . . . . . . . . . . . 3

S1.2 Δ-MLP . . . . . . . . . . . . . . . . . . . . . . . . . . . . . . . 4

S1.3 Validation . . . . . . . . . . . . . . . . . . . . . . . . . . . . . . 4

S1.3.1 Seed Dependence . . . . . . . . . . . . . . . . . . . . . . . . 5

S1.4 Architecture . . . . . . . . . . . . . . . . . . . . . . . . . . . . . 5

S1.4.1 Number of message passing layers and number of channels . . . . . . . . 6

S1.4.2 Cutoff . . . . . . . . . . . . . . . . . . . . . . . . . . . . . 6

S1.4.3 L0 vs L1 . . . . . . . . . . . . . . . . . . . . . . . . . . . . 6

**S2 Overall validation** **8**

S2.1 DFT to DFT validation: revPBE-D3 to r2SCAN; revPBE-D3 to PBE-D3 . . . . 8

S2.2 DFT to CCSD(T) validation: revPBE-D3 to CCSD(T); r$^2$SCAN to CCSD(T); PBE-D3 to CCSD(T) . . . . . . . . . . . . . . . . . . . . . . . . . . . 12

**S3 Electronic Structure-computational details** **14**

S3.1 CCSD(T) . . . . . . . . . . . . . . . . . . . . . . . . . . . . . . 14

S3.2 Validating local CCSD(T) approximations in MRCC and ORCA . . . . . . . . . 15

**S4 Molecular dynamics computational details** **15**

S4.1 Simulation details for classical molecular dynamics . . . . . . . . . . . . . . 15

S4.2 Simulation details for path integral molecular dynamics . . . . . . . . . . . . 16

S4.3 Self-Diffusion Coefficients . . . . . . . . . . . . . . . . . . . . . . . 16

S4.4 Density isobar . . . . . . . . . . . . . . . . . . . . . . . . . . . . 17

**S5 Additional results** **17**

S5.1 Classical results . . . . . . . . . . . . . . . . . . . . . . . . . . . 17

S5.2 Comparison to literature . . . . . . . . . . . . . . . . . . . . . . . 18

**S6 Reaching better data efficiency** **19**

S6.1 How many 5.5 Å clusters are actually needed? . . . . . . . . . . . . . . . . . . 19

S6.2 Can we make local CCSD(T) cheaper? . . . . . . . . . . . . . . . . . . . . . . 21

S6.3 What is the most cost efficient dataset? . . . . . . . . . . . . . . . . . . . . . 22

**References** **24**

Many of the SI tests are performed in the same self-consistent spirit as the convergence tests in the main text for computational efficiency, therefore for a given property, the final 'converged' result may not be the basis set converged CCSD(T) result. For clarity, the level of theory (CCSD(T) basis set/ local approximation thresholds) is given in the caption of each Figure. Furthermore, all tests on the RDF in the SI are done at the experimental density (0.997 g/cm$^3$ at 298 K) to enable consistent comparisons.

# S1 Machine learning

## S1.1 Dataset

The Δ-learning approach used in this work requires 2 machine learning potentials (MLPs) and therefore two datasets. The baseline MLP was trained on periodic boxes containing 126 waters, labeled with DFT forces and energies. The Δ-MLP was trained on gas phase clusters cut from periodic configurations and labeled with the energy difference between CCSD(T) and DFT. The final CCSD(T) MLP is given as a sum of the periodic baseline model plus the Δ-MLP. Note that any time we refer to the CCSD(T) MLP, we are referring to this total sum of the baseline plus Δ-MLPs. In practice, configurations for both model datasets were sampled from the same simulations and the datasets was generated over multiple generations.

An additional challenge comes from the extent of the differences between the DFT baseline and CCSD(T). For example, revPBE-D3 predicts a density roughly 10 % smaller than experiment[1] (the target for the CCSD(T) model), and therefore care should be taken to ensure sufficiently diverse density sampling to obtain stable reliable models, as well as sampling from both DFT and

CCSD(T) configurations.

In summary, the following simulations were sampled, with each used to provide reference configurations for both baseline and Δ-MLPs: NPT pressure scan at DFT level spanning both positive and negative pressures (specifically -1500, -500, -300, 1, 2, 500, 1000, 4000, 8000 bar). This gave an initial CCSD(T) MLP, which was then used to sample again NPT with the same positive and negative pressures, this time ensuring CCSD(T) level water structures were covered in the dataset. Finally to ensure the model could robustly describe NQEs, a final sampling of PIMD NVT simulations over a density scan (0.85, 0.9, 0.95, 1.0, 1.05 g/cm$^3$) was done to give the final CCSD(T) model.

## S1.2 Δ-MLP

One challenge with the Δ-learning strategy is the significantly reduced information content training on energies alone, with each configuration containing only a single energy. This is in contrast to the standard approach for fitting DFT-level MLPs, where energy gradients (forces) provide significant additional information on the slope of the potential energy surface. Therefore, we have specifically explored the number of clusters required to reliably predict bulk properties from gas phase clusters (as well as the size of clusters as discussed in the main text) and this is discussed more in Section S6.

The clusters were cleaved out from the same set of structures used for the periodic DFT dataset. An O atom was randomly selected within each structure (or its supercell depending on the cluster radius $r_c$), and all O atoms within $r_c$ around this O atom were selected. The cluster was then formed by then incorporating all H atoms bonded to the selected O atoms.

## S1.3 Validation

We have performed a series of validation tests on the models to test the completeness of the dataset, MLP architectures and seed and baseline dependencies. As in the main, we directly benchmark against condensed phase properties. For the majority of these tests, we show results for the density and radial distribution function (RDF), since reliably computing the diffusion

coefficient requires additional computational effort, which would be significant for the roughly one hundred models we have trained for validation purposes. Nevertheless, the extent of structuring of the RDF has been shown to be a good proxy for the water self-diffusion coefficient,[2] and therefore the properties we validate against still represent a highly thorough suite of tests. Moreover, testing directly on condensed phase observables makes a much more direct connection than the typical benchmarks performed on gas phase properties of small clusters.

### S1.3.1 Seed Dependence

To test the completeness of the dataset, we trained several Δ-MLP models (with the r$^2$SCAN baseline) with different train-test-validation splits as well as seeds. The resulting thermodynamic properties of the different seeds is shown in Figure S1. We find that all seeds correct the shortcomings of r$^2$SCAN with respect to experiment, with minor variations within 0.03 g/cm$^3$ on the density.

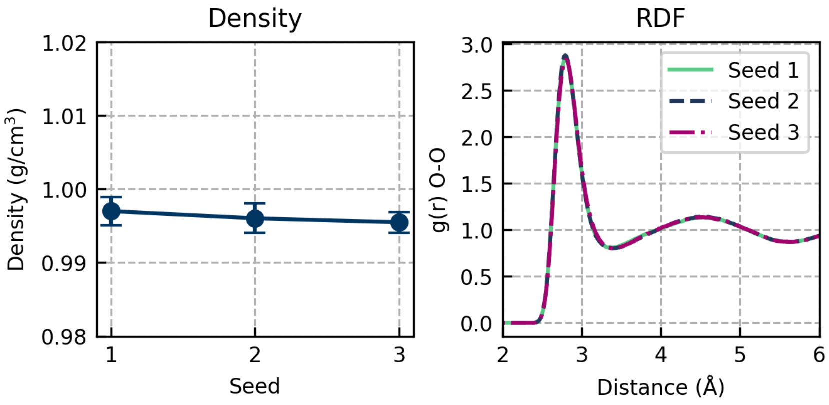


**Figure S1: Seed dependence.** Comparison of three different seeds for the Δ model on the CCSD(T) prediction of density and RDF. [Level of theory of CCSD(T): QZ/TightPNO]

## S1.4 Architecture

We use the MACE framework[3] to train MLPs for both the baseline and Δ-MLPs. Choosing hyperparameters for the baseline MLP are now routine and straightforward (and are detailed in the main Methods section) and the focus in this section are to find optimal hyperparameters for the Δ-MLP. In particular, previous work has highlighted that a shorter range cutoff may be

needed/sufficient to fit this potential,[4] which can help to lower the overall computational cost. Therefore the most prominent hyperparameter to consider is the cutoff, but we also consider the inclusion of 2 message-passing layers and the number of channels. The messages can also be chosen to be equivariant or invariant (L=0 or L=1).

#### S1.4.1 Number of message passing layers and number of channels

We first explore the effect of the number of message passing layers (1 or 2), with the number of channels (64 vs 128) and cutoff (4 vs 5 Å). To get a further handle on the variation, we perform this test using the three different baselines. We test the effect of these architectures on the density and RDF as a function of the (cumulative) cluster sizes in the training set as shown in Figure S2. In the limit of large clusters (7.5 Å), the predicted densities for all models agrees within error bars. However, for smaller cluster sizes at the sizes we are targeting for the final model (5.5 Å and smaller), the single layer model shows larger fluctuations for the $r^2$SCAN and PBE-D3 baselines, and so we chose the 2-layer, 64 channel model with a 4 Å cutoff for the final architecture.

#### S1.4.2 Cutoff

We show in Figure 2 in the main text, that the dataset containing up to 5.5 Å clusters reliably reproduces the limit of larger cluster sizes. For this 5.5 Å cluster dataset, we have also tested the effect of the MACE cutoff for the 2 layer, 64 channel model selected from previous section. We test cutoffs from 3.0 Å to 4.5 Å in steps of 0.5 Å, such that the complete receptive field of the first layer is filled. Figure S3 shows that while the RDF is already well described by the smallest receptive field, the density requires a larger cutoff of 4.0 Å.

#### S1.4.3 L0 vs L1

In Figure S4, we compare the effect of utilizing either invariant (L=0) or equivariant (L=1) messages for a MACE cutoff of 4.0 Å and 64 channels. We find that differences are minor for both density and RDF, highlighting that invariant messages are sufficient with our dataset.

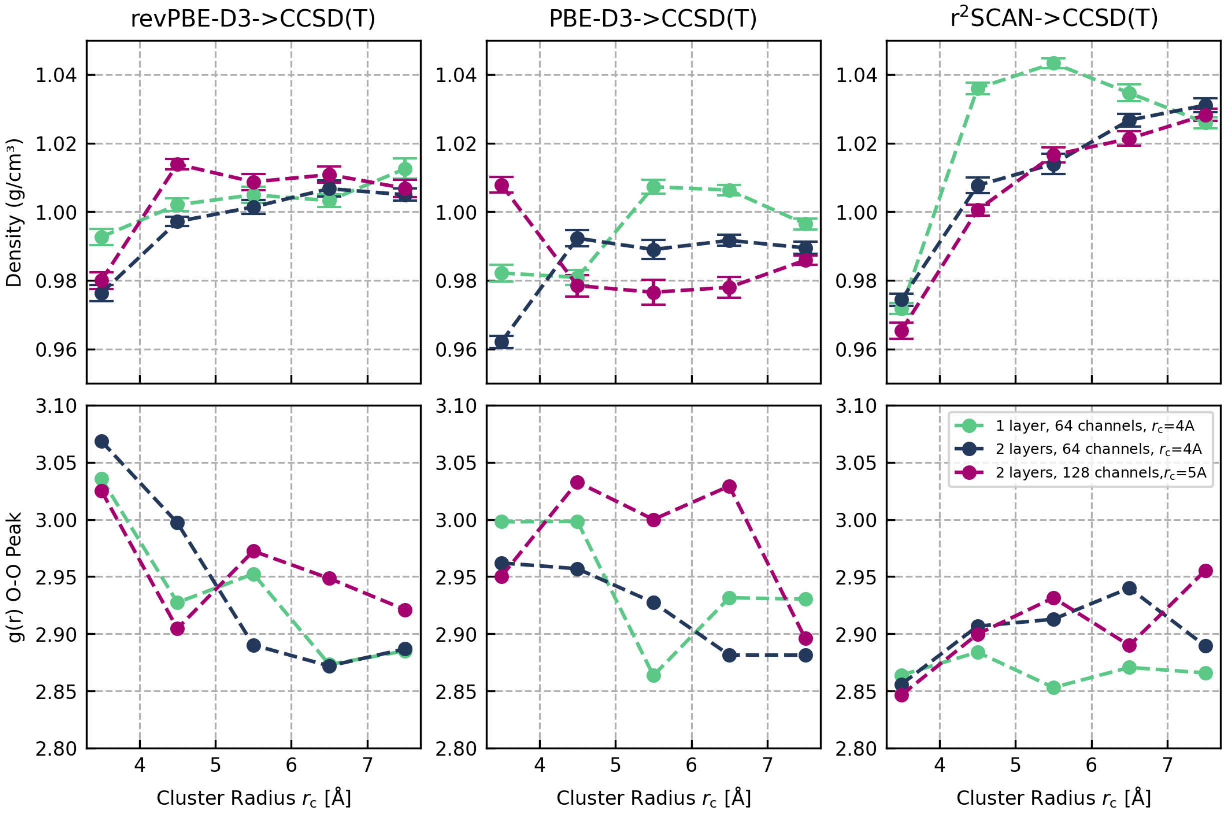


**Figure S2: Architecture and dataset relationship.** Comparison of density and RDF peak position for the three different DFT baselines for various MACE architectures for the Δ-MLP as a function of the largest size of clusters in the dataset. Both 1 and 2-layer models are shown, where we have also varied the number of channels and cutoff radius ($r_c$) for the 2-layer model. [Level of theory of CCSD(T): CBS(DZ/TZ) NormalLNO]

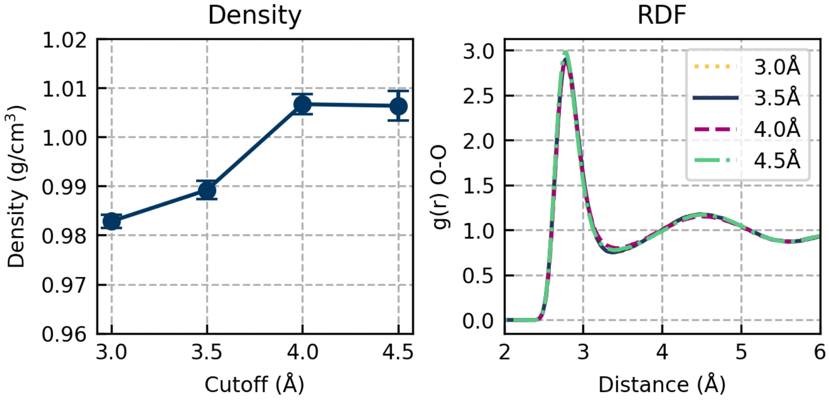


**Figure S3: Cutoff of Δ-MLP.** Effect of cutoff for 2 layer Δ model with 5.5 Å the maximum cluster radius in the dataset using revPBE-D3 as a baseline. [Level of theory of CCSD(T): QZ/TightPNO ]

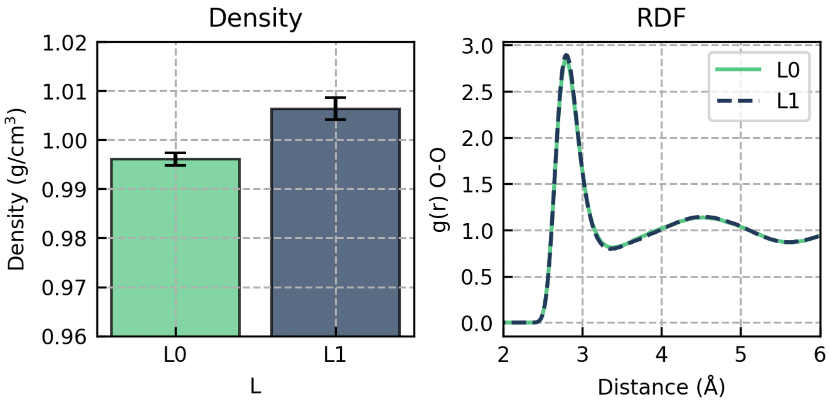


**Figure S4: Comparison of MACE message-passing type.** Effect of invariant (L=0) vs equivariant (L=1) messages for Δ-MLP on CCSD(T) density and RDF using revPBE-D3 as a baseline. [Level of theory of CCSD(T): QZ/TightPNO]

# S2 Overall validation

## S2.1 DFT to DFT validation: revPBE-D3 to r2SCAN; revPBE-D3 to PBE-D3

As an additional validation to the self-consistant convergence procedure described in the main text, here we validate the Δ-learning approach by showing that we can learn between different levels of DFT. In this validation, we can directly compare to the target periodic result, obtained from MLPs trained on periodic data. We have chosen three DFT functionals, which give varying predictions of physical properties as well as the different physical attributes (*i.e.*, the incorporation of dispersion corrections). Figures S5, S6 and S7 all show the Δ approach learns the difference between these different DFT levels for the O-O, H-H and O-H RDFs, reliably reproducing the reference periodic target. We note that there are slight differences between the revPBE-D3 to PBE-D3 model compared to the periodic PBE-D3 result. This may be a result of not having included PBE-D3 water structures in the dataset, as further discussed in Section S2.2 below.

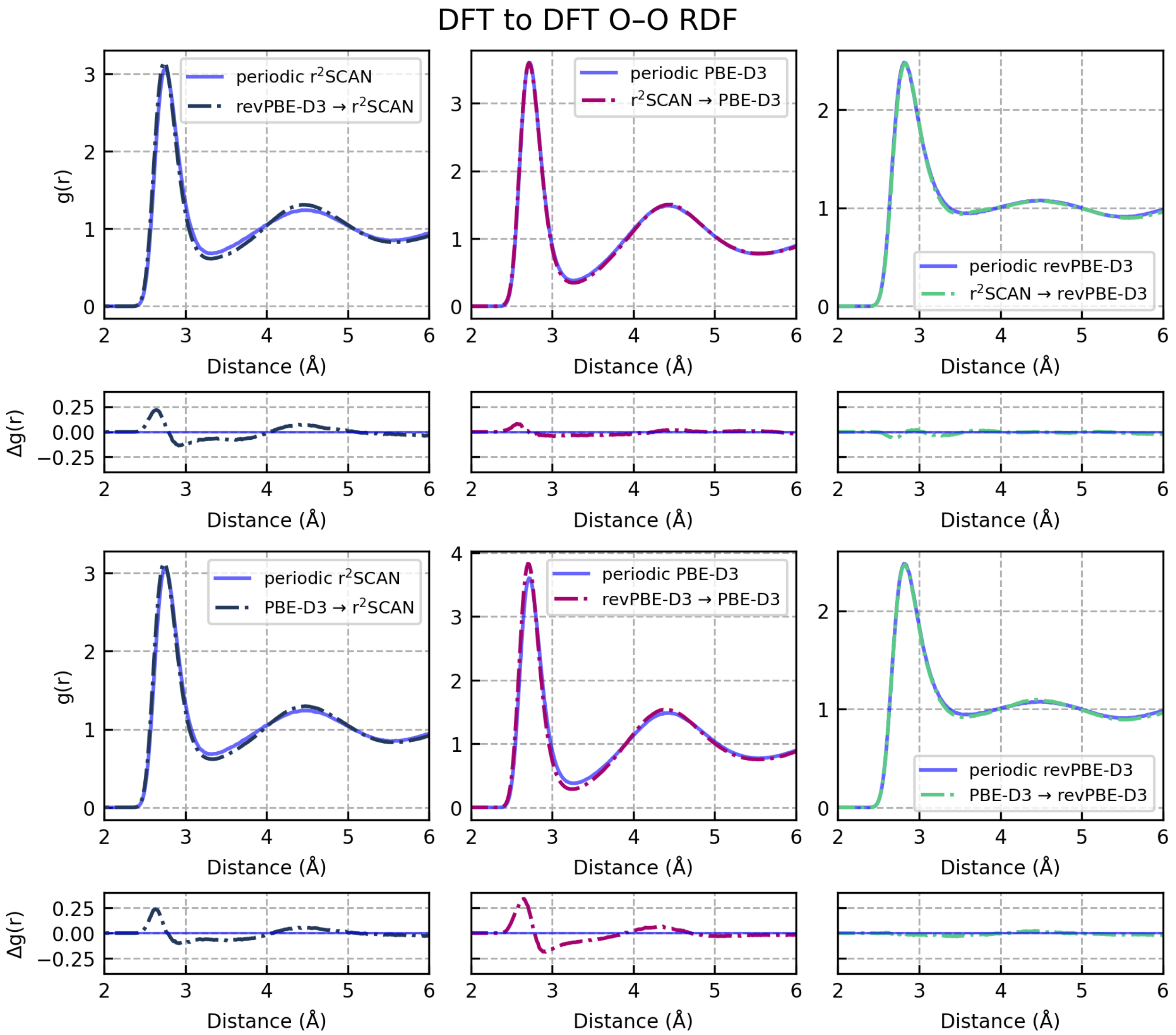


**Figure S5: DFT to DFT validation on the O-O RDF** O-O RDFs for all permutations going between r$^2$SCAN, revPBE-D3 and PBE-D3. Solid blue lines give the periodic model result, and dashed lines are the delta model with $A \rightarrow B$ corresponding to baseline $\rightarrow \Delta$ model . Differences between the delta and corresponding periodic model are given in the lower panel.

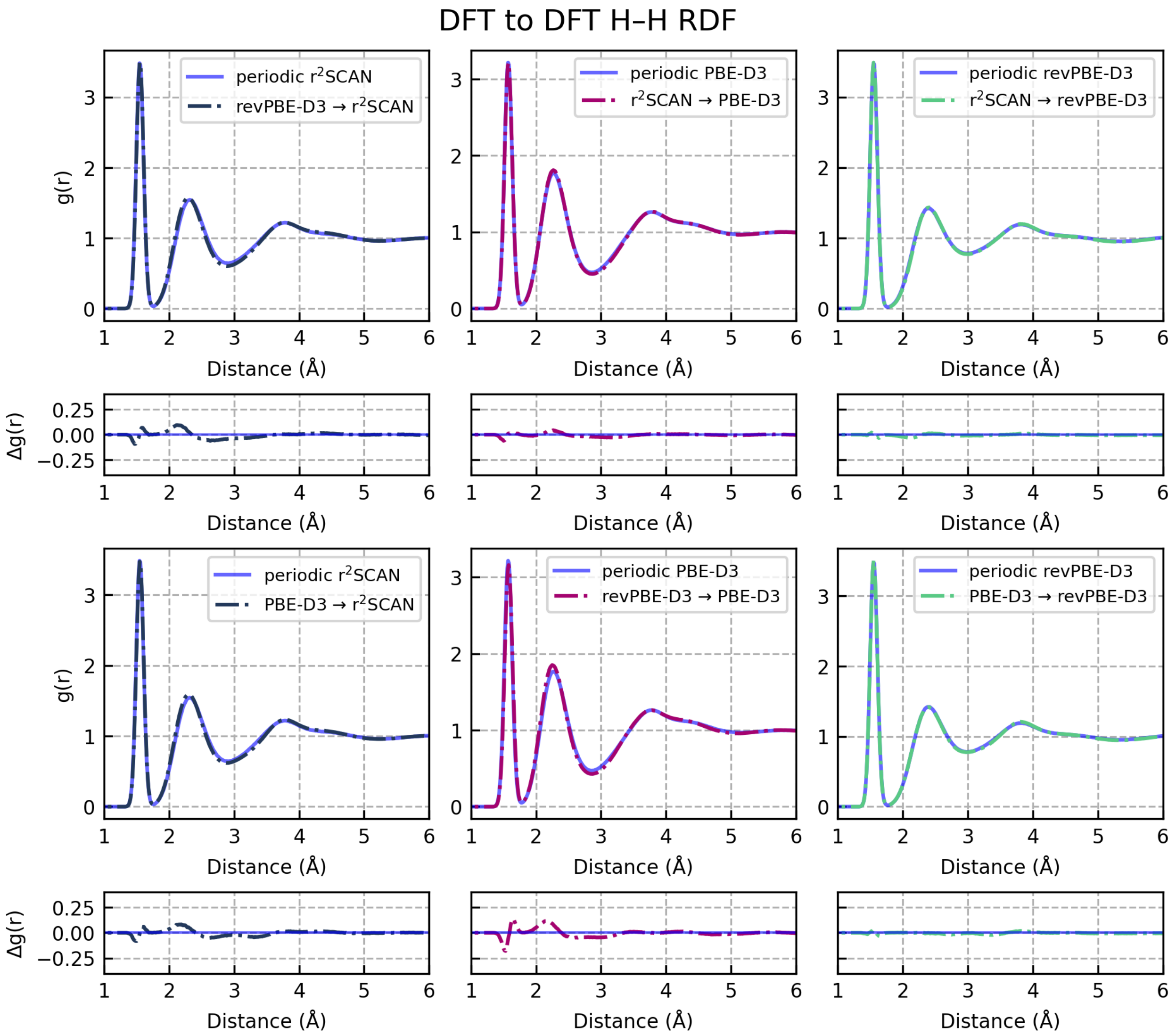


**Figure S6: DFT to DFT validation on the H-H RDF.** H-H RDFs for all permutations going between $r^2$SCAN, revPBE-D3 and PBE-D3. Solid blue lines give the periodic model result, and dashed lines are the delta model with $A \to B$ corresponding to baseline $\to \Delta$ model. Differences between the delta and corresponding periodic model are given in the lower panel.

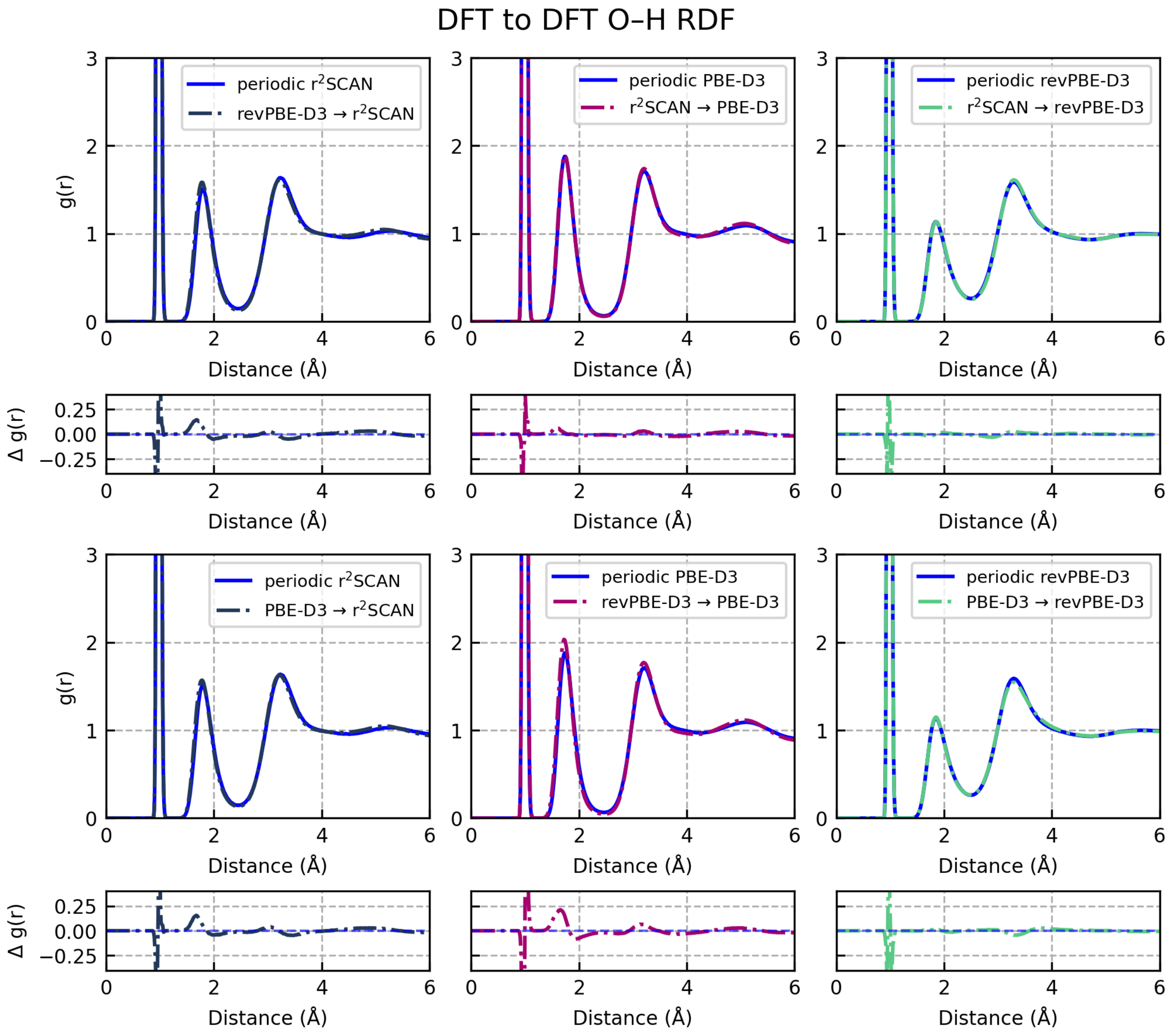


**Figure S7: DFT to DFT validation on the O-H RDF.** O-H RDFs for all permutations going between r$^2$SCAN, revPBE-D3 and PBE-D3. Solid blue lines give the periodic model result, and dashed lines are the delta model with $A \rightarrow B$ corresponding to baseline $\rightarrow \Delta$ model. Differences between the delta and corresponding periodic model are given in the lower panel.

## S2.2 DFT to CCSD(T) validation: revPBE-D3 to CCSD(T); $r^2$SCAN to CCSD(T); PBE-D3 to CCSD(T)

To further validate the reliability of the Δ learning approach to learn the CCSD(T) reference, Figures S8 and S9 shows the combined Δ-MLP predictions with different DFT baselines (revPBE-D3, $r^2$SCAN and PBE-D3). We show that both revPBE-D3 and $r^2$SCAN baselines are in excellent agreement for the RDFs and density, suggesting the robustness of our framework, since revPBE-D3 and $r^2$SCAN are very different, both in terms of functional design philosophy and also prediction of physical properties of liquid water. We also show that the RDF is least sensitive to the chosen baseline, with all three baselines in excellent agreement. The CCSD(T) MLP with PBE-D3 as a baseline underpredicts the density by ∼2 %. While this may indicate that PBE-D3 is inherently harder to learn from, it should also be noted that we did not explicitly include the DFT (PBE-D3) water structures in the Δ-MLP dataset. We have found that including explicitly both DFT and CCSD(T) water in both the periodic and Δ datasets resulted in more stable models. Nevertheless, this PBE-D3 result indicates that reasonable results can still be obtained from less judicious sampling. However, for the most quantitatively predictive models, care should be taken to ensure the datasets span both the baseline and target levels of theory.

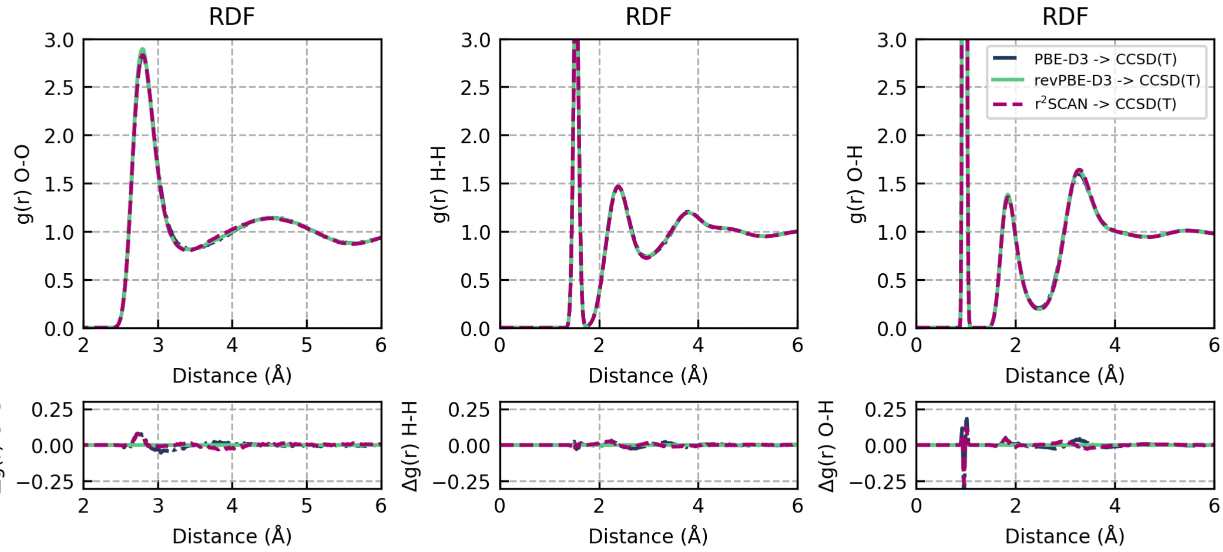


**Figure S8: DFT to CCSD(T) validation.** Predictions of CCSD(T) RDFs based on three DFT baselines (PBE-D3, $r^2$SCAN and revPBE-D3). [Level of theory of CCSD(T): QZ/ TightPNO]

In Figure S10, we plot violin plots of the distribution of the predicted forces on the baseline

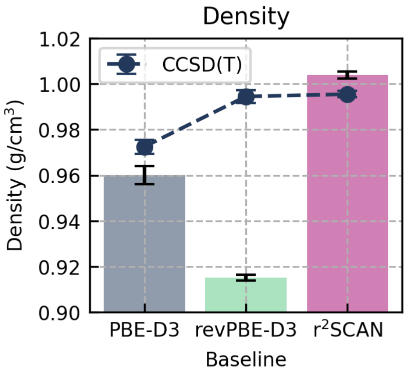


**Figure S9: DFT to CCSD(T) validation.** Predictions of CCSD(T) density (dark blue) based on three DFT baselines (PBE-D3, r$^2$SCAN and revPBE-D3). The corresponding baseline DFT density is shown in the barplot. [Level of theory of CCSD(T): QZ/ TightPNO]

dataset for the three Δ-MLPs. It can be seen that the Δ-MLP with an r$^2$SCAN baseline predicts much smaller forces, indicating that r$^2$SCAN predicts closer energies and forces to CCSD(T) and so the Δ-MLP has an easier learning task going between r$^2$SCAN to CCSD(T).

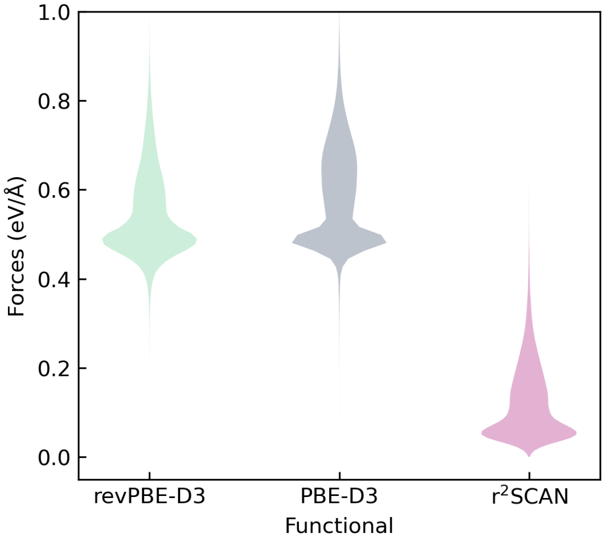


**Figure S10: Predicted force magnitude of the Δ-MLP.** Violin plot of the magnitude of the atomic forces predicted by the Δ-MLPs as a function of their baseline.

# S3 Electronic Structure-computational details

## S3.1 CCSD(T)

For completeness, here we repeat the CCSD(T) settings described in the Methods Section of the Main text. The CCSD(T) calculations, using local approximations, were performed on both the MRCC[5] and ORCA[6] program. We used the domain-based local pair natural orbital (DLPNO) approximation[7–9] within ORCA for the final simulations shown in the main text (Main: Figures 3 and 4 and Table 1). We used relatively conservative and accurate settings in ORCA, using the "TightPNO" DLPNO thresholds for the correlation energy calculations and turning off the RIJCOSX approximation for the Hartree-Fock calculations. For many of the tests, particularly when demonstrating the cluster size convergence in Figure 2 of the main text, when validating the use of local approximations in Section S3.2, and demonstrating computational cost savings from employing looser local approximation thresholds in Section S6, we use the local natural orbital (LNO) approximation[10,11] to CCSD(T) in MRCC. In particular, we utilize density-fitting with the HF calculations as well as the standard "normal" LNO thresholds (which we have dubbed "NormalLNO" throughout this SI).

We have used the Dunning family[12] of correlation consistent basis sets, where aug-cc-pV$X$Z was used on the O atom and cc-pV$X$Z was used on the H atom – dubbed jul-cc-pV$X$Z – with $X$ representing its size in terms of double (DZ), triple (TZ) or quadruple (QZ) zeta. We consider the CBS(DZ/TZ) and CBS(TZ/QZ), which involve a two-point complete basis set (CBS) extrapolation, using parameters taken from Neese and Valeev,[13] for the enclosed pair of basis functions. We use the def2-QZVPP-RI-JK auxiliary basis function for density-fitting/resolution-of-identity Hartree–Fock (HF) computations in MRCC, and the resolution-of-identity auxiliary basis sets from Weigend[14,15] corresponding to the AO basis sets for subsequent local CCSD(T) calculations in ORCA and MRCC.

## S3.2 Validating local CCSD(T) approximations in MRCC and ORCA

As discussed in Section S6, we have used relatively conservative DLPNO and HF settings for the ORCA calculations, while we considered cheaper settings, employing density-fitting in the HF calculations and only "normal" LNO thresholds, for the MRCC calculations. As we show in Figure S11, these less-conservative MRCC settings lead to observables which are in agreement with those from the conservative ORCA settings. This agreement validates the both flavors of local approximations used in MRCC (LNO) and ORCA (DLPNO). This sets the stage for using MRCC to study more complex systems, since its use of less conservative settings result in a computational cost is almost two orders of magnitude lower, as discussed in Section S6.

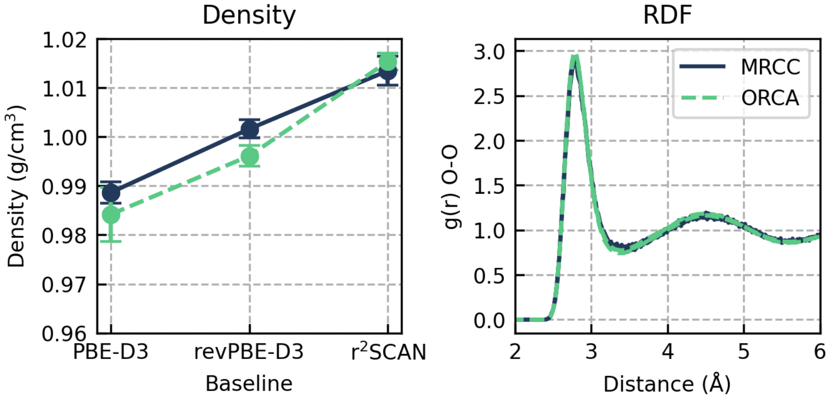


**Figure S11: Comparison between DLPNO and LNO CCSD(T).** Comparison of density and O-O RDF predictions for the CCSD(T) MLP using MRCC with `normal` LNO thresholds and density fitting in the HF for cbs(DZ/TZ) basis set for the Δ-MLP.

# S4 Molecular dynamics computational details

## S4.1 Simulation details for classical molecular dynamics

All classical simulations were performed using the Large-scale Atomic/Molecular Massively Parallel Simulator (LAMMPS) code, coupled with the `Symmetrix` library,[16] using the `symmetrix/mace` pair style in tandem with the `hybrid/overlay` pairstyle to sum the periodic and Δ-MLPs. All simulations boxes contained 126 waters and were performed at 298 K. Classical simulations used at 0.5 fs timestep. The density was obtained from simulations in the isothermal isobaric

ensemble (NPT) at a pressure of 1 bar, with a barostat relaxation time of 1 ps. All density simulations were run for at least 500 ps, with block averaging to obtain the errorbar. Radial distribution functions and diffusion coefficients were obtained from simulations in the canonical (NVT) ensemble. For all convergence tests, these were computed at the experimental density, with a box size of 15.577 Å to ensure consistancy. For the final model production simulations, the RDF and diffusion coefficient were obtained from simulations at the computed density from the NPT simulations. In all cases, the CSVR[17] thermostat was used, with a temperature of 298 K and a temperature relaxation time of 0.1 ps.

## S4.2 Simulation details for path integral molecular dynamics

Nuclear quantum effects were approximately described via ring polymer molecular dynamics simulations, using the i-Pi code coupled with LAMMPS and `Symmetrix`. For the density, an initial simulation was performed in the NPT ensemble for 400 ps using 32 beads at 298 K and 0.25 fs timestep. A Langevain thermostat with a $\tau$=100 fs, with the PILE thermostat used for the barostat with $\tau$=10 fs. 9 independent replicas were then sampled from this initial simulation and run for between 300 and 400 ps each. The final density was given by the average and standard error over these independent simulations. To obtain the RDFs and diffusion coefficient, an initial T-RPMD simulation was performed in the NVT ensemble at the above computed density, with 32 beads and a 0.25 fs timestep for 150 ps. A PILE thermostat with $\lambda$=0.5 and $\tau$=100 fs was used. 9 independent configurations were then sampled from this simulation and run for 100 ps each. The diffusion coefficient was then computed from the average of the individual diffusion coefficients of the centroid of the ring polymers as described in further detail in the next section. RDFs were obtained from the positions trajectory of a bead of the ring polymer.

## S4.3 Self-Diffusion Coefficients

The self-diffusion coefficient was obtained from fitting the slope of the mean squared displacement of the water oxygen atoms vs time from 2 to 20 ps. The diffusion coefficient obtained for a 126 water simulation box $D(L)$ was corrected for the finite size effects from using a periodic simulation

cell of length $L$ using the Yeh and Hummer correction:[18]

$$D(L) = D(\infty) - \zeta \frac{k_B T}{6\pi\eta L} \quad (1)$$

where $\zeta$ was taken as the experimental sheer viscosity of 0.8925 mPas and the numerical coefficient for a cubic simulation cell $\zeta$ as 2.837297. Table S1 summarises the classical and quantum diffusion coefficients with and without finite size correction:

Table S1: **Comparison of diffusion coefficient to experiment.** Summary of density and diffusion coefficient from classical and PIMD simulations at 298 K compared to experiment. Diffusion coefficients show both the finite size corrected value ($D(\infty)$) and before finite size correction ($D(L)$). Error bars are shown in parentheses.

| | $\rho$ [g/cm$^3$] | $D(\infty)$ [Å$^2$/ps] | $D(L)$ [Å$^2$/ps] |
|---|---|---|---|
| Experiment | 0.997 | 0.23 | – |
| CCSD(T) (quantum) | 0.989 (0.003) | 0.22 (0.01) | 0.18 |
| CCSD(T) (classical) | 0.991 (0.0001) | 0.21 (0.01) | 0.17 |

### S4.4 Density isobar

Classical simulations spanning 250K - 330K in increments of 10K were performed to resolve the density isobar at 1 bar. For each temperature, eight independent replicas were simulated, for at least 3 ns per replica. Error bars were obtained from the standard error of the independent replicas.

## S5 Additional results

### S5.1 Classical results

Figure S12 shows the RDFs obtained from classical MD simulations from the CCSD(T), where the corresponding results including NQEs are given in Figure 3 in the main text.

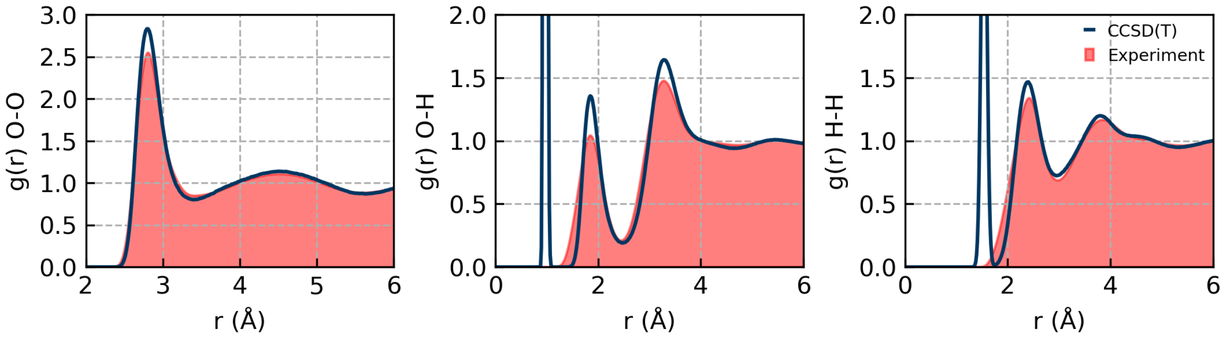


**Figure S12: Classical CCSD(T) structure of water.** CCSD(T) RDFs for liquid water at 298 K from classical MD simulations compared to experiment

## S5.2 Comparison to literature

Figure S13 compares the O-O RDF predicted by our final Δ-learned CCSD(T) model to previous CCSD(T) models. We compare to both previous MLP models from Daru *et al.*[19] and Chen *et al.*,[2] as well as models based on the many-body expansion, MB-pol[20] and q-AQUA-pol.[21] Overall, there is good agreement among all of the models, with some slight differences in the height of the first peak.

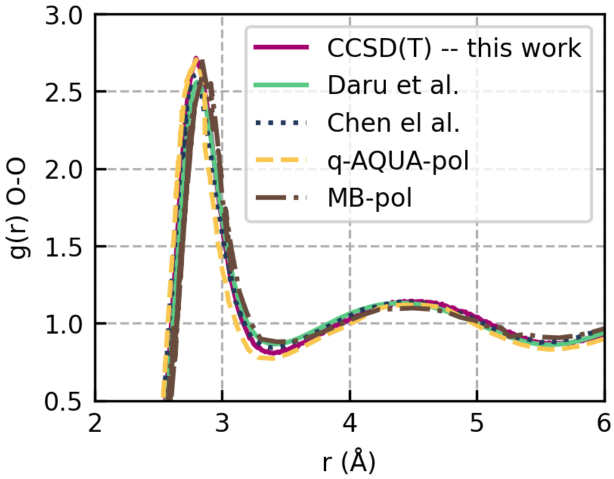


**Figure S13: Comparison to previous CCSD(T) models.** Comparison of our CCSD(T) O-O RDF from PIMD simulations at 298 K with literature CCSD(T) models. Daru *et al.* comes from PIMD simulations at 298 K from Reference.[19] Chen *et al.* comes from PIMD simulations at 300 K from.[2] q-AQUA-pol comes from PIMD simulations at 298 K from Reference.[21] MB-pol comes from classical simulations at 298 K from Reference.[22]

# S6 Reaching better data efficiency

As mentioned in the Discussion of the main text, the total cost to train the final dataset using conservative ORCA settings dataset with the jul-cc-pVQZ basis set is about 1.6 million CPUh. We give the rough breakdown of this total cost for each water cluster radius in Table S2. The majority of the cost (1.4 million CPUh) comes from the largest cluster radius of 5.5 Å. This consists of 1764 calculations in total, each of which costs roughly 800 CPUh. As we will show in the next two subsections, both the number of calculations as well as the cost for each cluster can be lowered significantly (by an order of magnitude each), which can substantially lower the cost of this dataset at no loss in accuracy.

Table S2: **Computational cost of full dataset with TightPNO DLPNO approximation.** A computational cost breakdown for the final dataset when using "TightPNO" DLPNO-CCSD(T) in ORCA with the jul-cc-pVQZ basis set. The calculations were performed on a mix of 96-core AMD Genoa nodes and 48-core Intel Cascadelake nodes, with 1,500 GB and 756 GB or RAM, respectively.

| Cluster radius (Å) | 2.5 | 3.5 | 4.5 | 5.5 | Total |
|---|---|---|---|---|---|
| Total cost (CPUh) | 204 | 29910 | 167460 | 1380056 | 1577630 |
| Average number of water molecules | 1.0 | 6.2 | 13.0 | 23.4 | |
| Number of clusters | 1814 | 1810 | 1810 | 1764 | 7198 |
| Average cost per cluster (CPUh) | 0.1 | 16.5 | 92.5 | 782.3 | |

We have compared our estimates of the costs to that by Daru *et al.*,[19] which has been described[4] to require "3,000 DLPNOCCSD(T) and over 13,000 DLPNO-MP2 for $(H2O)_{64}$ clusters". It has been described in Ref. 19 that 109 and 166 days were needed for 10,000 single-point DLPNO-MP2 and DLPNO-CCSD(T) calculations, respectively, using a total of 40 nodes (each with 20 cores). The resulting cost (in CPUh) for the work of Daru *et al.* is thus 191.5 days on 40 nodes, amounting to $\sim$3.7 million CPUh.

## S6.1 How many 5.5 Å clusters are actually needed?

As we will demonstrate in this section, we have generated a relatively conservative dataset for the final CCSD(T) model, and much fewer 5.5 Å clusters are actually needed than what we included within our final dataset in Table S2. In Figure S14, we plot the convergence of the final

CCSD(T) model for the density and RDF as a function of the number of randomly selected 5.5 Å clusters included within the dataset. This incorporates all the smaller clusters, so the point at 0 represents the cumulative 4.5 Å dataset in Figure 1 of the main text. We considered three different DFT baselines and it can be seen that beyond 100 clusters, the density is converged to within 0.01 g/cm$^3$ and 0.05 for the PBE-D3 and revPBE-D3 baseline. This leads to a 5 times overall decrease in computational cost to 275,808 CPUh, as shown in Table S3. There appears to be more challenges converging the r$^2$SCAN baseline, potentially arising from the lack of long-range dispersion interactions in the functional.

Table S3: **Computational cost of compact dataset with TightPNO DLPNO approximation.** A computational cost breakdown for the compact dataset when using "TightPNO" DLPNO-CCSD(T) in ORCA with the jul-cc-pVQZ basis set. The calculations were performed on a mix of 96-core AMD Genoa nodes and 48-core Intel Cascadelake nodes, with 1,500 GB and 756 GB or RAM, respectively. The cost for 100 5.5 Å clusters was scaled based on the total cost for the 1764 clusters within the full dataset.

| Cluster radius (Å) | 2.5 | 3.5 | 4.5 | 5.5 | Total |
|---|---|---|---|---|---|
| Total cost (CPUh) | 204 | 29910 | 167460 | 78234 | 275808 |
| Number of clusters | 1814 | 1810 | 1810 | 100 | 5534 |

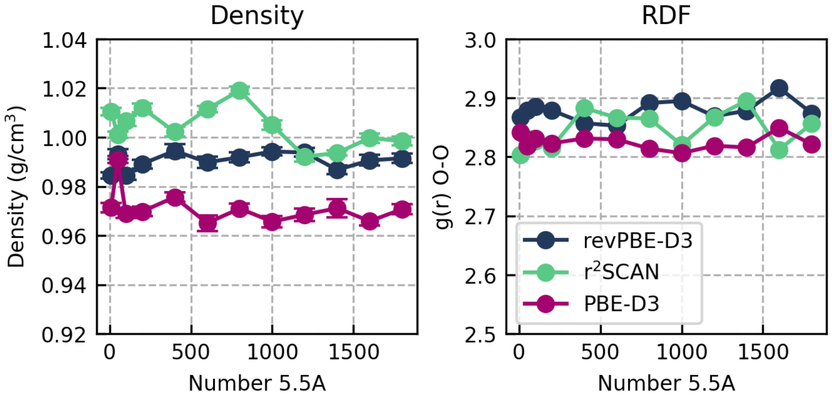


**Figure S14: Number of 5.5 Å clusters required in dataset.** Comparison of density and RDF for the CCCSD(T) MLP as a function of the number of 5.5 Å clusters added to the total cumulated dataset, with 0 corresponding to the cumulative 4.5 Å dataset. [CCSD(T) level of theory: QZ/TightPNO]

It is instructive to highlight that the small number of 5.5 Å clusters required to reach convergence arises from our choice of using a cumulative dataset. For example, in Figure S15, we show the convergence as a function of the number of 5.5 Å clusters, where the dataset only include 5.5 Å clusters, excluding the smaller clusters from the dataset. The convergence is less monotonic

compared to when the smaller clusters were included in the dataset. While the density converges to within 0.01 g/cm$^3$ within 500 clusters, ∼1500 clusters were needed to converge both the RDF and diffusion coefficient to 0.01 and $0.1\times10^{-9}$m$^2$/s.

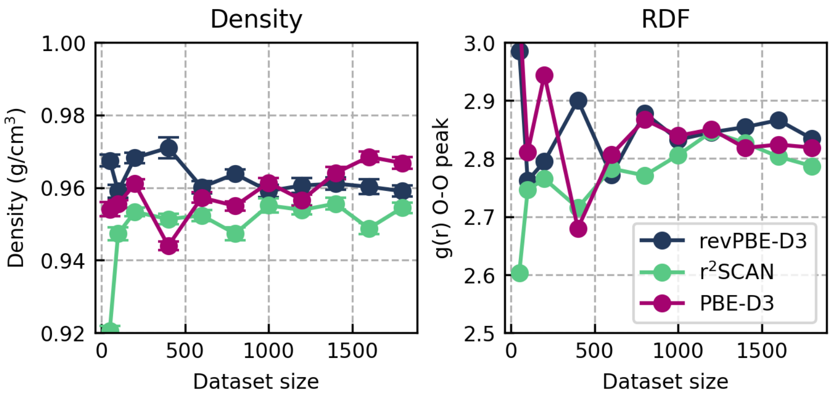


**Figure S15: Convergence with dataset size of non-cumulative dataset.** Comparison of density, and O-O RDF peak height as a function of total dataset size, where the dataset contains exclusively 5.5 Å radius clusters. [CCSD(T) level of theory: QZ/TightPNO]

Besides a much slower convergence with dataset size, the convergence as a function of cluster size also becomes more challenging using a non-cumulative dataset compared to a cumulative one (see Figure 1 of the main text). In Figure S16, it can be seen that while the density converges as a function of cluster size, the RDF becomes challenging to converge. For example, with all three baselines, the RDF peak appears to deivate going towards larger cluster sizes beyond 5.5 Å, probably arising because they require more than 1800 clusters to converge with a non-cumulative dataset.

## S6.2 Can we make local CCSD(T) cheaper?

We utilized conservative electronic structure parameters within ORCA as too loose approximations, particularly for the local approximations, can incur noise within the data that can affect the quality of the resulting MLP. This consists of turning off the RIJCOSX approximation as it was highlighted by Daru *et al.* to bring errors in the forces. Furthermore, we used the "TightPNO" set of thresholds for the DLPNO approximation. As shown in Table S2, these settings led to an overall cost of 1.6 million CPUh. While this is already less than previous works, it would become more routine if the cost can be decreased further. In Table S4, we show that the total cost for the

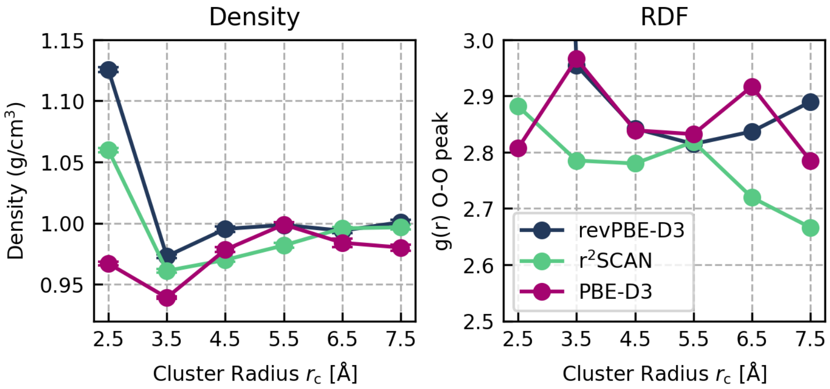


**Figure S16: Convergence with cluster size of non-cumulative datasets.** Comparison of CCSD(T) density and O-O RDF peak height for models trained on non-cumulative datasets, where a dataset with cluster radius N does not contain clusters of size N-1, N-2 etc. [CCSD(T) level of theory: CBS(DZ/TZ)/NormalLNO]

complete dataset can be lowered by one order of magnitude if we utilize the LNO approximation in MRCC together with less conservative "normal" thresholds.

Table S4: **Computational cost of full dataset with "normal" LNO approximation.** A computational cost breakdown for the final dataset when using LNO-CCSD(T) in MRCC with the jul-cc-pVQZ basis set and "normal" LNO thresholds. The calculations were performed on 48-core Intel Cascadelake nodes with 756 GB or RAM.

| Cluster radius (Å) | 2.5 | 3.5 | 4.5 | 5.5 | Total |
|---|---|---|---|---|---|
| Total cost (CPUh) | 19 | 1504 | 10318 | 51570 | 63411 |
| Average number of water molecules | 1.0 | 6.2 | 13.0 | 23.6 | |
| Number of clusters | 1814 | 1814 | 1810 | 1798 | 7236 |
| Average cost per cluster (CPUh) | 0.0 | 0.8 | 5.7 | 28.7 | |

## S6.3 What is the most cost efficient dataset?

We consolidate all of the observations made within this section and find that combining the more data-efficient training set together with the less conservative LNO-CCSD(T) settings gives a total dataset cost of 15,000 CPUh (see Table S5 and Figure S17 for a further breakdown). The resulting cost is more than 100× cheaper than the original dataset and electronic structure settings in Table S2. Furthermore, it is important to also highlight the low computational requirements. In fact, the efficient dataset can be computed all on a personal (32-core) desktop within the matter of three weeks. For example, the average amount of memory – typically the limiting factor for

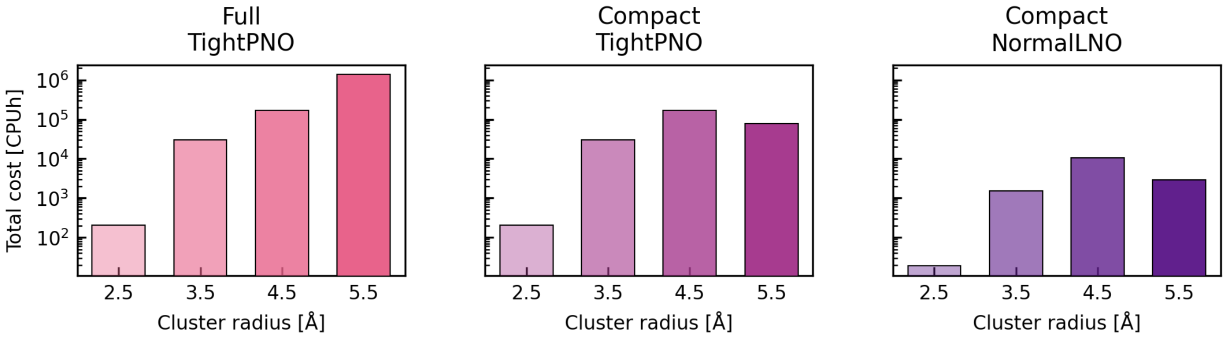


**Figure S17: Cost breakdown for dataset size and cluster radius.** Breakdown of cost for the total number of each cluster size for the full and compact datasets with both TightPNO and NormalLNO settings.

CCSD(T) calculations – for the 5.5 Å cluster calculations was 17 GB, with a maximum of 50 GB for the most expensive calculation (see Figure S18). These memory requirements can be easily met with commodity hardware and it is possible to further lower these memory requirements by storing more arrays to the disk using the `usedisk` parameter within MRCC.

Table S5: **Computational cost of compact dataset with "normal" LNO approximation.** A computational cost breakdown using the efficient dataset together with LNO-CCSD(T) in MRCC using the jul-cc-pVQZ basis set and "normal" LNO thresholds. The calculations were performed on 48-core Intel Cascadelake nodes with 756 GB or RAM.

| Cluster radius (Å) | 2.5 | 3.5 | 4.5 | 5.5 | Total |
|---|---|---|---|---|---|
| Total cost (CPUh) | 19 | 1504 | 10318 | 2868 | 14710 |
| Number of clusters | 1814 | 1814 | 1810 | 100 | 5538 |

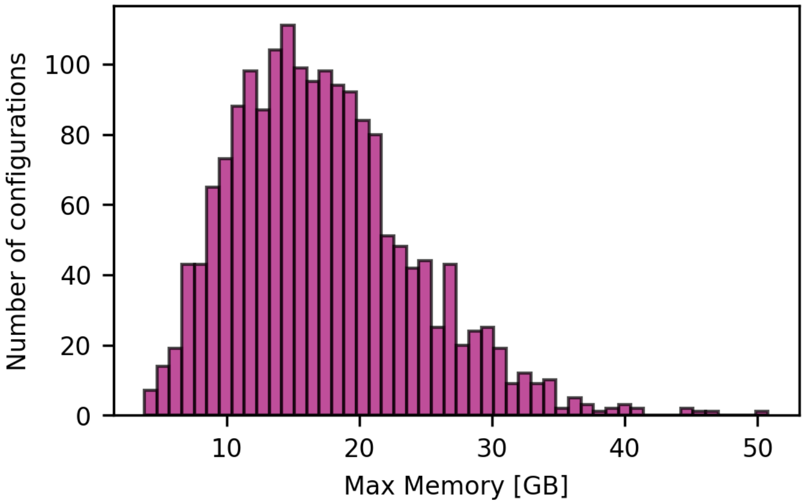


**Figure S18: Memory usage breakdown.** A histogram of the maximum memory for each 5.5 Å cluster calculation using the jul-cc-pVQZ basis set for "normal" LNO-CCSD(T).

# References


(1) Montero de Hijes, P.; Dellago, C.; Jinnouchi, R.; Kresse, G. Density Isobar of Water and Melting Temperature of Ice: Assessing Common Density Functionals. The Journal of Chemical Physics **2024**, 161, 131102.

(2) Chen, M. S.; Lee, J.; Ye, H.-Z.; Berkelbach, T. C.; Reichman, D. R.; Markland, T. E. Data-Efficient Machine Learning Potentials from Transfer Learning of Periodic Correlated Electronic Structure Methods: Liquid Water at AFQMC, CCSD, and CCSD(T) Accuracy. Journal of Chemical Theory and Computation **2023**, 19, 4510–4519.

(3) Batatia, I.; Kovacs, D. P.; Simm, G.; Ortner, C.; Csanyi, G. MACE: Higher Order Equivariant Message Passing Neural Networks for Fast and Accurate Force Fields. Advances in Neural Information Processing Systems. 2022; pp 11423–11436.

(4) Mészáros, B. B.; Szabó, A.; Daru, J. Short-Range Δ-Machine Learning: A Cost-Efficient Strategy to Transfer Chemical Accuracy to Condensed Phase Systems. Journal of Chemical Theory and Computation **2025**, 21, 5372–5381.

(5) Kállay, M.; Nagy, P. R.; Mester, D.; Rolik, Z.; Samu, G.; Csontos, J.; Csóka, J.; Szabó, P. B.; Gyevi-Nagy, L.; Hégely, B.; Ladjánszki, I.; Szegedy, L.; Ladóczki, B.; Petrov, K.; Farkas, M.;

Mezei, P. D.; Ganyecz, Á. The MRCC Program System: Accurate Quantum Chemistry from Water to Proteins. The Journal of Chemical Physics **2020**, 152, 074107.

(6) Neese, F.; Wennmohs, F.; Becker, U.; Riplinger, C. The ORCA Quantum Chemistry Program Package. The Journal of Chemical Physics **2020**, 152, 224108.

(7) Riplinger, C.; Neese, F. An Efficient and near Linear Scaling Pair Natural Orbital Based Local Coupled Cluster Method. The Journal of Chemical Physics **2013**, 138, 034106.

(8) Riplinger, C.; Sandhoefer, B.; Hansen, A.; Neese, F. Natural Triple Excitations in Local Coupled Cluster Calculations with Pair Natural Orbitals. The Journal of Chemical Physics **2013**, 139, 134101.

(9) Riplinger, C.; Pinski, P.; Becker, U.; Valeev, E. F.; Neese, F. Sparse Maps—A Systematic Infrastructure for Reduced-Scaling Electronic Structure Methods. II. Linear Scaling Domain Based Pair Natural Orbital Coupled Cluster Theory. The Journal of Chemical Physics **2016**, 144, 024109.

(10) Nagy, P. R.; Samu, G.; Kállay, M. Optimization of the Linear-Scaling Local Natural Orbital CCSD(T) Method: Improved Algorithm and Benchmark Applications. Journal of Chemical Theory and Computation **2018**, 14, 4193–4215.

(11) Nagy, P. R.; Kállay, M. Approaching the Basis Set Limit of CCSD(T) Energies for Large Molecules with Local Natural Orbital Coupled-Cluster Methods. Journal of Chemical Theory and Computation **2019**, 15, 5275–5298.

(12) Peterson, K. A.; Dunning, T. H. Accurate Correlation Consistent Basis Sets for Molecular Core–Valence Correlation Effects: The Second Row Atoms Al–Ar, and the First Row Atoms B–Ne Revisited. The Journal of Chemical Physics **2002**, 117, 10548–10560.

(13) Neese, F.; Valeev, E. F. Revisiting the Atomic Natural Orbital Approach for Basis Sets: Robust Systematic Basis Sets for Explicitly Correlated and Conventional Correlated Ab Initio Methods? Journal of Chemical Theory and Computation **2011**, 7, 33–43.

(14) Weigend, F.; Häser, M.; Patzelt, H.; Ahlrichs, R. RI-MP2: Optimized Auxiliary Basis Sets and Demonstration of Efficiency. Chemical Physics Letters **1998**, 294, 143–152.

(15) Hellweg, A.; Hättig, C.; Höfener, S.; Klopper, W. Optimized Accurate Auxiliary Basis Sets for RI-MP2 and RI-CC2 Calculations for the Atoms Rb to Rn. Theoretical Chemistry Accounts **2007**, 117, 587–597.

(16) Witt, W. C. Symmetrix, Available at: https://github.com/wcwitt/symmetrix. 2025.

(17) Bussi, G.; Donadio, D.; Parrinello, M. Canonical Sampling through Velocity Rescaling. The Journal of Chemical Physics **2007**, 126, 014101.

(18) Yeh, I.-C.; Hummer, G. System-Size Dependence of Diffusion Coefficients and Viscosities from Molecular Dynamics Simulations with Periodic Boundary Conditions. The Journal of Physical Chemistry B **2004**, 108, 15873–15879.

(19) Daru, J.; Forbert, H.; Behler, J.; Marx, D. Coupled Cluster Molecular Dynamics of Condensed Phase Systems Enabled by Machine Learning Potentials: Liquid Water Benchmark. Physical Review Letters **2022**, 129, 226001.

(20) Palos, E.; Bull-Vulpe, E. F.; Zhu, X.; Agnew, H.; Gupta, S.; Saha, S.; Paesani, F. Current Status of the MB-pol Data-Driven Many-Body Potential for Predictive Simulations of Water Across Different Phases. Journal of Chemical Theory and Computation **2024**, 20, 9269–9289.

(21) Qu, C.; Yu, Q.; Houston, P. L.; Conte, R.; Nandi, A.; Bowman, J. M. Interfacing Q-AQUA with a Polarizable Force Field: The Best of Both Worlds. Journal of Chemical Theory and Computation **2023**, 19, 3446–3459.

(22) Gartner, T. E. I.; Hunter, K. M.; Lambros, E.; Caruso, A.; Riera, M.; Medders, G. R.; Panagiotopoulos, A. Z.; Debenedetti, P. G.; Paesani, F. Anomalies and Local Structure of Liquid Water from Boiling to the Supercooled Regime as Predicted by the Many-Body MB-pol Model. The Journal of Physical Chemistry Letters **2022**, 13, 3652–3658.
